# Prevalence of respiratory viruses using polymerase chain reaction in children with wheezing, a systematic review and meta–analysis

**DOI:** 10.1371/journal.pone.0243735

**Published:** 2020-12-14

**Authors:** Cyprien Kengne–Nde, Sebastien Kenmoe, Abdou Fatawou Modiyinji, Richard Njouom

**Affiliations:** 1 National AIDS Control Committee, Epidemiological Surveillance, Evaluation and Research Unit, Yaounde, Cameroon; 2 Department of Virology, Centre Pasteur of Cameroon, Yaoundé, Cameroon; 3 Faculty of Sciences, Department of Animals Biology and Physiology, University of Yaoundé I, Yaoundé, Cameroon; Queen's University Belfast, UNITED KINGDOM

## Abstract

**Introduction:**

Wheezing is a major problem in children, and respiratory viruses are often believed to be the causative agent. While molecular detection tools enable identification of respiratory viruses in wheezing children, it remains unclear if and how these viruses are associated with wheezing. The objective of this systematic review is to clarify the prevalence of different respiratory viruses in children with wheezing.

**Methods:**

We performed an electronic in Pubmed and Global Index Medicus on 01 July 2019 and manual search. We performed search of studies that have detected common respiratory viruses in children ≤18 years with wheezing. We included only studies using polymerase chain reaction (PCR) assays. Study data were extracted and the quality of articles assessed. We conducted sensitivity, subgroup, publication bias, and heterogeneity analyses using a random effects model.

**Results:**

The systematic review included 33 studies. Rhinovirus, with a prevalence of 35.6% (95% CI 24.6–47.3, I^**2**^ 98.4%), and respiratory syncytial virus, at 31.0% (95% CI 19.9–43.3, I^**2**^ 96.4%), were the most common viruses detected. The prevalence of other respiratory viruses was as follows: human bocavirus 8.1% (95% CI 5.3–11.3, I^**2**^ 84.6%), human adenovirus 7.7% (95% CI 2.6–15.0, I^**2**^ 91.0%), influenza virus6.5% (95% CI 2.2–12.6, I^**2**^ 92.4%), human metapneumovirus5.8% (95% CI 3.4–8.8, I^**2**^ 89.0%), enterovirus 4.3% (95% CI 0.1–12.9, I^**2**^ 96.2%), human parainfluenza virus 3.8% (95% CI 1.5–6.9, I^**2**^ 79.1%), and human coronavirus 2.2% (95% CI 0.6–4.4, I^**2**^ 79.4%).

**Conclusions:**

Our results suggest that rhinovirus and respiratory syncytial virus may contribute to the etiology of wheezing in children. While the clinical implications of molecular detection of respiratory viruses remains an interesting question, this study helps to illuminate the potential of role respiratory viruses in pediatric wheezing.

**Review registration:**

PROSPERO, CRD42018115128.

## Introduction

Wheezing is a common health challenge in early childhood.^1^ More than one–third of children aged < 2 years experience at least one episode of wheezing, and one–fifth experience recurrent wheezing [1]. Pediatric wheezing can sometimes be severe enough to justify hospitalization, admission to intensive care units, and mechanical ventilation [2,3]. Studies show that children with a history of lower respiratory tract infections, primarily bronchiolitis, have an increased risk of developing transient wheezing up to age 13 [4,5]. Wheezing may additionally be related to the development of asthma in adulthood [4,6].

Wheezing is believed to be induced by viral infection [7,8]. Common viruses associated with wheezing include rhinovirus (RV), human respiratory syncytial virus (HRSV), human metapneumovirus (HMPV), human parainfluenza virus (HPIV), enterovirus (EV), human adenovirus (HAdV), human bocavirus (HBoV), human coronavirus (HCoV), and influenza virus [9,10].

Traditional respiratory viral diagnostic methods, such as cell culture and serology, have limitations. For example, RV is difficult to isolate by cell culture [11,12], and the large number of RV serotypes poses a major challenge for serological assays [13]. Compared to molecular detection, traditional diagnostic assays have a lower sensitivity for all common respiratory viruses [14,15]. These traditional diagnostics, however, have commonly been used to document respiratory viral prevalence, particularly in early literature [10]. Consequently, initial studies may have over or understated the role of certain respiratory viruses in pediatric wheezing. Molecular detection has revealed that RV may play a substantive role in the clinical manifestation of respiratory disease than original thought [16,17]. Additionally, molecular detection helped to identify the presence of new viruses such as HMPV and HBoV in children with respiratory signs [18,19].

Thus, the prevalence of common respiratory viruses in wheezing still remains unclear. Assessing the impact of the upcoming HRSV vaccination will require reliable data on prevalence [20]. Accurate viral prevalence data could contribute to optimizing and controlling antibiotic use, and providing better guidance for therapeutic decision making. The aim of this systematic review is to synthesize the prevalence of respiratory viruses in wheezing children, from publications that use molecular tools.

## Methods

### Study design

The study was performed according to the preferred reporting items for systematic reviews and meta–analyses(PRISMA) guidelines (S1 Table) [21]. Ethical approval was not required because the study does not involve the inclusion of humans and/or animals. The present study was registered in the PROSPERO database (PROSPERO: CRD42018115128;https://www.crd.york.ac.uk/prospero/display_record.php?RecordID=115128).

### Inclusion criteria

We included clinical trial, cohort, case–control, and cross–sectional studies that reported the prevalence of respiratory viruses in children ≤18 years presenting with wheezing. The definitions of wheezing were adapted as described by the authors of the primary studies. In the case of duplicate studies, where the same population was recruited and examined during the same period, only the most recent or complete study was included.

### Exclusion criteria

Reviews, letters to the editor, studies with interrupted study periods, and studies of patients with underlying medical conditions such as bronchopulmonary dysplasia, cystic fibrosis and bronchiolitis obliterans were excluded. We also excluded studies that used non–PCR based methods for viral detection such as culture, time–resolved fluoroimmunoassay, enzyme immunoassay or immunofluorescence. Included articles with multiple follow up time were used only once for each virus.

### Search strategy

Relevant studies were identified through research conducted in Pubmed and Global Index Medicus. We used search terms associated with the common respiratory viruses including RV, EV, HRSV, HMPV, HPIV, HCoV, HAdV, HBoV, and influenza and with the clinical signs of wheezing. The search string used in Pubmed and Global Index Medicus is illustrated in S2 Table. The search was carried out without any language restrictions and considered publication dates until 01 July 2019. We used Google Translate for articles written in languages other than English and French. Study reference sections and relevant review articles were used to identify additional articles.

### Study selection

Two investigators (SK and AFM) independently selected eligible studies based on the titles and abstracts from the list of references on the Rayyan website, a free web–based application used to assist authors of systematic reviews (https://rayyan.qcri.org/welcome) [22]. The selection process was summarized in a PRISMA flow chart [21].

### Data extraction

The complete versions of the selected articles were downloaded and reviewed by two authors (AFM and SK) of the study. The data were extracted using a pre–designed abstraction form, specifically we collected: study design, study country, World Health Organization (WHO) region, sampling method, mean age of study participants, percentage of male patients, total number of patients tested, and total number of positive samples for each virus.

### Appraisal of methodological quality and risk of bias

Two authors (AFM and SK) independently assessed the quality of each study using the Hoy et al assessment scale [23]. This scale has 10 dichotomous questions that assess the internal and external validity of a study (S3 Table). According to these questions, articles were classified as low, moderate, or high risk of bias.

### Data synthesis and analysis

Disagreements in the selection of studies, data extraction and evaluation of study quality were resolved by discussion and consensus among the authors. Inter rater agreement for study selection was calculated using the kappa statistic Kappa values [–1–0], [0–0.2], [>0.2–0.4], [>0.4–0.6], [>0.6–0.8] and [>0.8–1] represented an extremely weak, very weak, weak, medium, satisfactory and excellent inter–rater agreement respectively [24]. Data were analyzed using the 'meta' package of the statistical software R (version 3.5.1) [25,26]. Unadjusted prevalence has been recalculated based on the information of crude numerators and denominators provided by individual studies. Prevalence was reported with a 95% confidence interval (CI) and a 95% prediction interval (PI). The variance of the study specific prevalence was stabilized with the Freeman–Tukey dual arcsine transformation before pooling the data within a random–effects meta–analysis model [27]. Egger's test served to assess the presence of publication bias [28]. A p–value of <0.10 for the Egger test was considered statistically significant. Heterogeneity was evaluated by the χ^2^ test on Cochrane's Q statistic [29], which was quantified by H and I^2^ values. The I^2^ statistic estimates the percentage of total variation across studies due to true between–study differences rather than chance. In general, I^2^ values >70% indicate the presence of substantial heterogeneity [30]. Subgroup analyses were performed for the following subgroups: WHO region, mean age group (0–2 years, 0–5 years, and 0–18 years), and a p–value <0.05 was considered statistically significant. The effect of variables that could explain the heterogeneity in the included studies was examined by a univariate and multivariate metaregression model. To assess the influence of design and quality of included studies an overall estimated sensitivity analysis was performed using only cross-sectional studies and studies with low risk of bias.

## Results

### Study selection and characteristics

**Fig 1:**
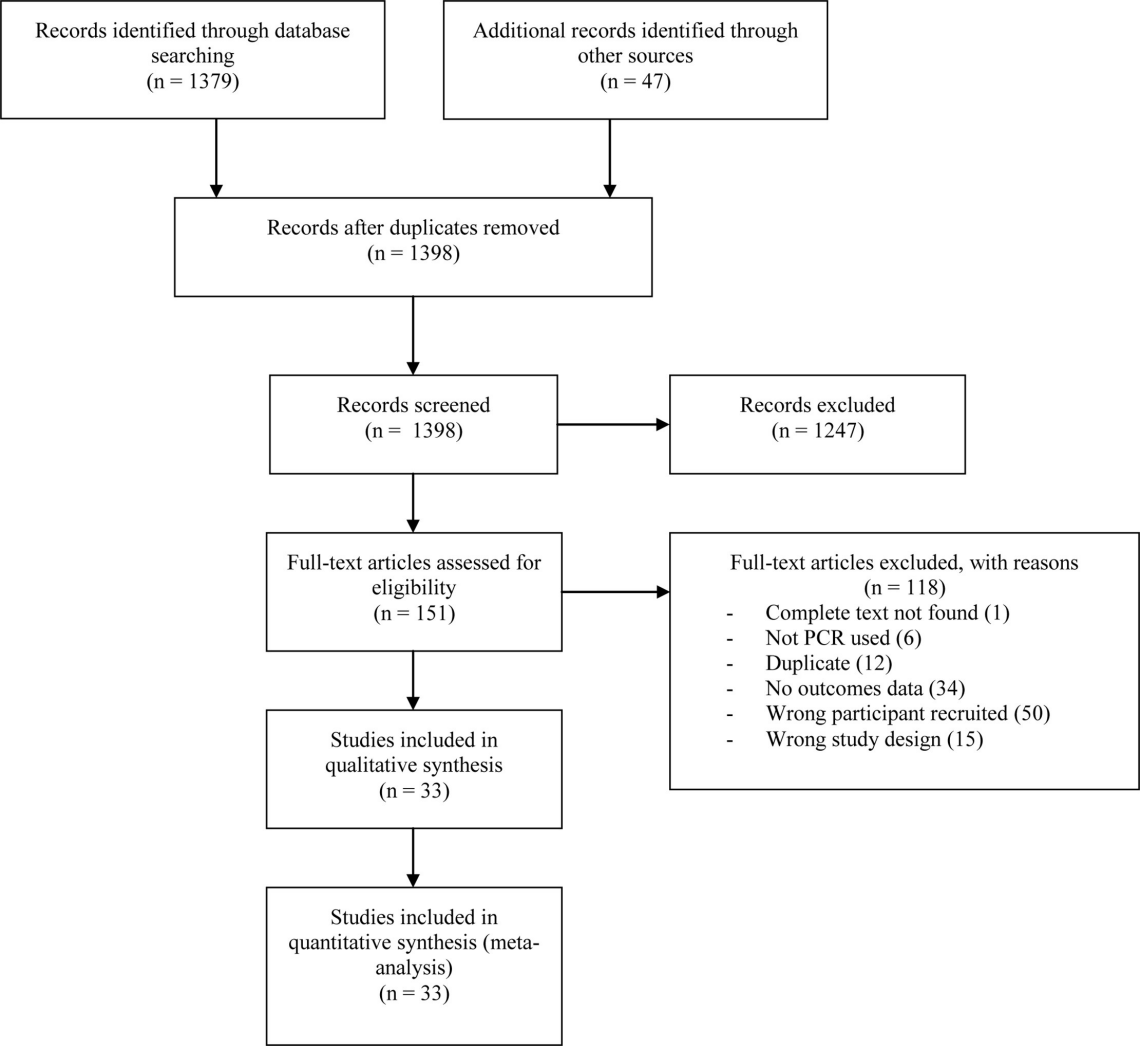
PRISMA flow chart of literature search and selection process.

Initially, we identified 1426 publication abstracts and removed 28 duplicates (Fig 1). After screening, we excluded 1247 that we found to be irrelevant, most of which were describing patients with asthma, bronchiolitis, atopy, chronic obstructive pulmonary disease, pneumonia or underlying medical conditions; studies on therapy, vaccination, prophylaxis, immune response or animals; and reviews. We assessed the texts of the remaining 151 papers for eligibility, of which 118were excluded most often because respiratory viruses were not investigated in the study (25;21.2%), wheezing patients were not recruited (47;39.8%), or it was an ineligible study type (15;12.7%)(S4 Table). Study selection inter rater agreement was κ = 0.87, representing excellent interrater agreement. Thirty–three full texts were retained for the review and included in the meta–analysis [31–63].

### Characteristics of included studies

Study participants were recruited between January 1992 and November 2014 (Table 1). According to the Shapiro-Wilk test, the age distribution did not follow a normal distribution (p <0.001) and therefore we expressed the age as the median and interquartile range. The cumulative number of samples tested from study participants was 18,365; however, the number of participants tested for each virus was variable. The percentage of males in each study ranged from 50–75%. The majority of participants recruited in included studies (20, 60.6%) were aged <5 years. The studies were published between 2002 and 2018. Most included studies were performed by consecutive sampling (25; 75.8%), had a prospective recruitment (29; 87.9%), had a moderate risk of bias (19; 57.6%), and tested specimens from the nasopharynx (29; 87.9%). Individual characteristics of included studies are presented in S5 Table.

**Table 1:** Sociodemographic and clinical characteristics of included studies.

Characteristics	N = 33
%Male. range	50.1–75
Age (years)	Mean: 1.5 (IQR: [1.0–3.2])
Age range	
• < 2 years	10 (30%)
• < 5 years	10 (30%)
• 0–18 years	9 (27%)
• Unclear/Not described	5 (15%)
Period of inclusion of participants. range	Jan/1992-Nov/2014
Year of publication. range	2002–2018
Study design	
• Case control	1 (3%)
• Clinical trial	7 (21%)
• Cohort	9 (27%)
• Cross-sectional	16 (48%)
Sampling	
• Consecutive	25 (75%)
• Random	8 (24%)
Timing of data collection	
• Prospective	29 (87%)
• Retrospective	4 (12%)
Study bias	
• Low risk	14 (42%)
• Moderate risk	19 (57%)
WHO region	
• Africa	2 (6%)
• America	3 (9%)
• Eastern Mediterranean	1 (3%)
• Europe	15 (45%)
• South-East Asia	2 (6%)
• Western Pacific	10 (30%)
Clinical presentation	
• Acute wheezing	30 (91%)
• Recurrent wheezing	3 (9%)
Sample type	
• Nasal samples	11 (33%)
• Oropharyngeal samples	4 (13%)
• Nasopharyngeal samples	29 (88%)
• Tracheal samples	2 (6%)

### Prevalence of viral infections among children with wheezing

**Fig 2:**
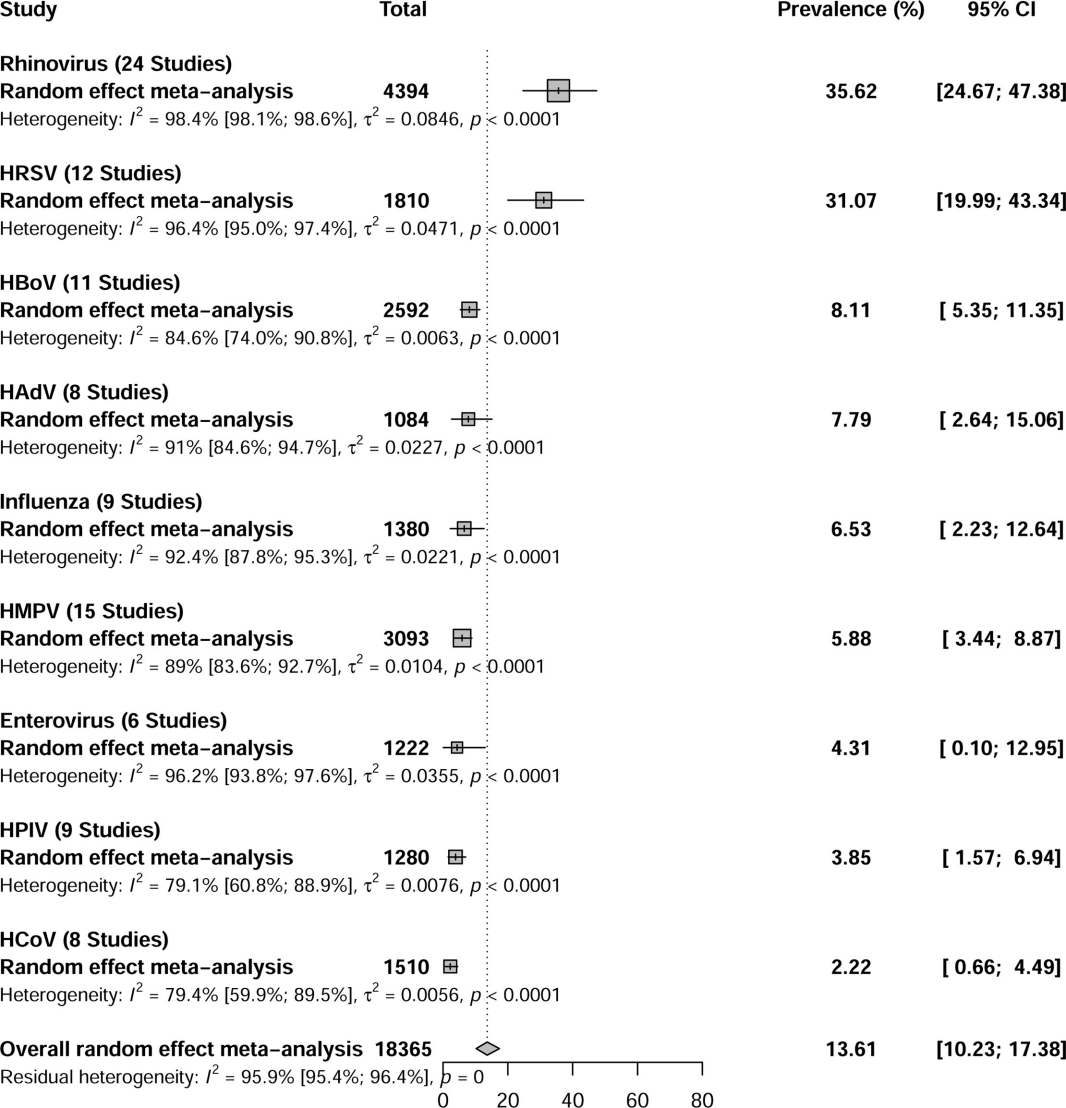
Global prevalence of respiratory viruses among children aged < 18 years with wheezing, January 1992-November 2014.

The prevalence of viruses detected with molecular assays in children with wheezing was: RV 35.6% (95% CI 24.6–47.3,I^2^ 98.4%), HRSV 31.0% (95% CI 19.9–43.3,I^2^ 96.4%), HBoV 8.1% (95% CI 5.3–11.3, I^2^ 84.6%), HAdV 7.7% (95% CI 2.6–15.0, I^2^ 91.0%),influenza virus 6.5% (95% CI 2.2–12.6,I^2^ 92.4%),HMPV 5.8% (95% CI 3.4–8.8, I^2^ 89.0%), EV 4.3% (95% CI 0.1–12.9, I^2^96.2%), HPIV 3.8% (95% CI 1.5–6.9, I^2^ 79.1%), and HCoV 2.2% (95% CI 0.6–4.4, I^2^ 79.4%) (Fig 2 and S1–S9 Figs). When sensitivity analyses were conducted for risk of bias and study design, they did not differ from the overall analysis (Table 2). Considerable heterogeneity was present in overall prevalence and sensitivity analysis for all viruses. Publication bias was present for studies that examined HMPV, HPIV, and HCoV (S10–S18 Figs).

**Table 2:** Overall meta-analysis for global prevalence of respiratory viral infections in children with wheezing.

	Prevalence. % (95%CI)	95% Prediction interval	N Studies	N Participants	H (95%CI)	I^2^ (95%CI)	P heterogeneity	P Egger test
**Rhinovirus**								
Overall	35.6 [24.6–47.3]	[0.0–90.9]	24	4394	7.8 [7.1–8.5]	98.4 [98.1–98.6]	< 0.001	0.632
Low risk	30.3 [14.0–49.7]	[0.0–96.2]	11	1974	9.6 [7.6–9.7]	98.7 [98.3–99.0]	< 0.001	0.932
Cross sectional studies	30.0 [16.5–45.5]	[0.0–86.7]	10	1659	6.3 [5.4–7.4]	97.5 [96.6–98.2]	< 0.001	0.882
**HRSV**								
Overall	31.0 [19.9–43.3]	[0.3–79.5]	12	1810	5.2 [4.4–6.1]	96.4 [95.0–97.4]	< 0.001	0.481
Low risk	29.3 [15.3–45.6]	[0.0–86.7]	8	1395	6.0 [4.9–7.2]	97.2 [96.0–98.1]	< 0.001	0.552
Cross sectional studies	20.6 [9.6–34.3]	[0.0–73.6]	6	931	4.0 [3.1–5.4]	94.0 [89.6–96.6]	< 0.001	0.996
**HBoV**								
Overall	8.1 [5.3–11.3]	[0.7–21.4]	11	2592	2.5 [1.9–3.3]	84.6 [74.0–90.8]	< 0.001	0.677
Low risk	6.8 [1.6–14.8]	[0.0–59.0]	4	953	3.5 [2.4–5.2]	92.1 [83.0–96.3]	< 0.001	0.799
Cross sectional studies	7.4 [4.6–10.8]	[0.6–19.8]	8	1539	2.0 [1.4–2.9]	76.3 [52.6–88.1]	< 0.001	0.248
**HAdV**								
Overall	7.7 [2.6–15.0]	[0.0–40.1]	8	1084	3.3 [2.5–4.3]	91.0 [84.6–94.7]	< 0.001	0.734
Low risk	11.6 [3.6–23.1]	[0.0–63.3]	5	822	3.4 [2.4–4.9]	91.8 [83.9–95.8]	< 0.001	0.565
Cross sectional studies	9.9 [2.4–21.0]	[0.0–58.9]	6	888	3.8 [2.8–5.1]	93.2 [87.9–96.2]	< 0.001	0.672
**Influenza**								
Overall	6.5 [2.2–12.6]	[0.0–35.4]	9	1380	3.6 [2.8–4.6]	92.4 [87.8–95.3]	< 0.001	0.142
Low risk	8.3 [1.6–19.0]	[0.0–61.4]	5	965	4.3 [3.2–5.8]	94.7 [90.4–97.1]	< 0.001	0.184
Cross sectional studies	6.4 [0.6–16.6]	[0.0–59.0]	5	861	3.9 [2.9–5.4]	93.7 [88.2–96.6]	< 0.001	0.226
**HMPV**								
Overall	5.8 [3.4–8.8]	[0.0–21.2]	15	3093	3.0 [2.4–3.6]	89.0 [83.6–92.7]	< 0.001	0.001
Low risk	4.7 [2.4–7.6]	[0.0–16.2]	5	1086	1.7 [1.0–2.7]	65.7 [10.4–86.9]	0.020	0.071
Cross sectional studies	7.7 [4.2–11.9]	[0.0–25.7]	9	1612	2.7 [2.0–3.5]	86.4 [76.2–92.2]	< 0.001	0.049
**Enterovirus**								
Overall	4.3 [0.1–12.9]	[0.0–49.9]	6	1222	5.1 [4.0–6.5]	96.2 [93.8–97.6]	< 0.001	0.808
Low risk	5.8 [0.2–16.8]	[0.0–64.7]	5	1107	5.4 [4.1–7.0]	96.6 [94.3–98.0]	< 0.001	0.658
Cross sectional studies	0.4 [0.0–1.2]	NA	2	654	1.0	0.0	0.380	NA
**HPIV**								
Overall	3.8 [1.5–6.9]	[0.0–17.0]	9	1280	2.1 [1.6–3.0]	79.1 [60.8–88.9]	< 0.001	0.081
Low risk	5.5 [1.2–12.2]	[0.0–39.4]	5	865	2.9 [2.0–4.2]	88.2 [75.0–94.4]	< 0.001	0.125
Cross sectional studies	3.8 [0.9–8.3]	[0.0–24.1]	6	931	2.3 [1.6–3.4]	82.5 [63.0–91.7]	< 0.001	0.143
**HCoV**								
Overall	2.2 [0.6–4.4]	[0.0–12.1]	8	1510	2.2 [1.5–3.0]	79.4 [59.9–89.5]	< 0.001	0.025
Low risk	1.6 [0.0–4.4]	[0.0–17.2]	5	1107	2.4 [1.6–3.6]	83.2 [61.8–92.6]	< 0.001	0.179
Cross sectional studies	1.7 [0.0–5.7]	[0.0–98.0]	3	869	2.7 [1.6–4.6]	87.0 [62.9–95.4]	< 0.001	0.128

CI: Confidence interval; RV: Rhinovirus; HCoV: Human Coronavirus; HPIV: Human Parainfluenzavirus; HMPV: Human Metapneumovirus; HRSV: Human Respiratory Syncytial Virus; HAdV: Human Adenovirus; HBoV: Human Bocavirus; EV: Enterovirus; NA: Not applicable.

### Subgroup analysis and metaregression

All subgroup analyses are presented in S6 Table. There were significant differences between age group and respiratory virus prevalence for RV (p = 0.024), HAdV (p< 0.001), influenza (p = 0.001) and EV (p = 0.012). Rhinovirus (> 2 year), HAdV (>2 years) and EV (> 2 years) were associated with older ages while influenza (< 1 year) was associated with younger ages. RV (p< 0.001), influenza (p = 0.016), HMPV (p< 0.001), and EV (p = 0.042) prevalence varied significantly according to WHO region. The prevalence of viruses was significantly different according to the detection assay used for RV (<0.001), HBoV (p = 0.001) and HCoV (p = 0.007). Compared to conventional RT-PCR, real-time RT-PCR was associated with higher prevalences for HCoV while conventional RT-PCR was associated with higher prevalences for RV and HBoV. There were no differences for the remaining subgroup analyses. Substantial heterogeneity was present most subgroup analyses. The multivariate metaregression model (S7 Table) indicates that the detection technique contributed to the heterogeneity of the results for the prevalence of RV, HRSV, Influenza and HBoV. The age of the participants contributed to the heterogeneity of the results for the prevalence of HAdV, Influenza and HCoV. In agreement with the Egger test and visual inspection of the funnel plot, there was evidence of publication bias for some age subgroups and WHO region for RV, HRSV, HBoV, HMPV and HCoV (S6 Table and S10–S18 Figs).

## Discussion

This study is the first systematic review of respiratory viruses in children with wheezing for nearly 30 years. The findings emphasize the strong association between the detection of RV and HRSV in wheezing children. Deoxyribonucleic acid viruses, HBoV and HAdV, were the second most commonly detected viruses. This study also showed a preponderance of RV, HAdV, and EV in older children and influenza in younger children.

Contrary to common knowledge that RV is associated with asymptomatic infection and mild illness, RV could have an important role in the clinical presentation of pediatric wheezing. As previously reported by the narrative reviews on this topic [7,10,11,64]. RV and HRSV were the most common viruses found in patients with wheezing. Although RVs are not sensitive to all cell lines, and earlier research studies lacked the molecular tools available today, the quantitative review analysis done by Pattemore et al in 1996 also recognized the role of RV in developing wheezing [11]. The hypothesis of the importance of RV in respiratory infections is well supported by two recent systematic reviews [65,66]. One has shown that RV was the predominant virus in asthma which is usually accompanied by wheezing [65]. The second review recently demonstrated that RV infections in the first 3 years of life were significantly associated with a high risk of subsequent wheezing and asthma at pre–school age [66]. In contrast to the Pattemore et al. study, a low prevalence of HPIV has been observed in this review. This could be explained by the inclusion of antibody–detecting studies and the wide range of clinical definitions in the Pattemore et al [11]. The detection of antibodies in the studies represents both acute and past infections and thus reports the highest prevalence [3,67].

Many studies have shown that HRSV is a major causative agent of wheezing in children under 2 years of age [9,68–70]. Although not significant in our study, the prevalence of HRSV was inversely proportional to child age.

These findings must be considered within the context of study limitations, including a small number of studies that met inclusion criteria, few studies that described wheezing type, and a limited worldwide geographic representation. Wheezing is considered bronchiolitis in children <1 year of age [71]. Almost all of the studies included in this review have children <1 year of age that we cannot exclude and could therefore be the source of additional heterogeneity. The studies included in this systematic review reported the prevalence of EV and RV individually. It is, however, known that EV and RV cross react in real-time PCR assays and this should be taken into account while interpreting the study results [72,73]. Seasonality and study duration could also drive the variability in the prevalence of respiratory viruses. This study, however, did not take these into account because the prevalence data were not always reported by these parameters. Molecular detection alone cannot implicate the etiology of clinical signs, as asymptomatic carriage of some respiratory viruses is possible. For example, the causal role of HBoV in acute respiratory infections, which has been reported in asymptomatic infections and mostly reported in codetection, has been widely controversial to date [74]. Additionally, combinations of viruses detected, or co–detections were beyond the scope of this work.

The present study has multiple strengths, particularly the fact that many respiratory viruses were considered in the analysis and that only studies with molecular viral detection were included, a gold standard and common diagnostic tool for respiratory viruses. In addition, the methodological approach strengthened this analysis, with the inclusion of analyses for explanation of sources of heterogeneity and publication bias. We used a comprehensive search strategy and two independent authors were involved in all stages.

RV and HRSV were the predominant viruses most commonly detected in children with wheezing. HRSV was largely present in children ≤ 2 years. This systematic review also highlights the important role played by newly described viral agents, HBoV and HMPV, in wheezing.

The completion and marketing of an HRSV vaccine as well as the development of antivirals against respiratory viruses, especially for RV and HRSV, could greatly help to reduce the burden of wheezing in children. Preventive measures for HRSV should be directed to at risk populations, such as children ≤ 2 years. While molecular detection may illuminate which viruses are associated with pediatric wheezing, greater attention is needed to understand if and how these viruses cause wheezing in children.

## Supporting information

S1 Fig Global prevalence of rhinovirus in people with wheezing disorders. (PDF)

S2 Fig Global prevalence of human respiratory syncytial virus in people with wheezing disorders. (PDF)

S3 Fig Global prevalence of human bocavirus in people with wheezing disorders. (PDF)

S4 Fig Global prevalence of adenovirus in people with wheezing disorders. (PDF)

S5 Fig Global prevalence of influenza in people with wheezing disorders. (PDF)

S6 Fig Global prevalence of human metapneumovirus in people with wheezing disorders. (PDF)

S7 Fig Global prevalence of enterovirus in people with wheezing disorders. (PDF)

S8 Fig Global prevalence of human parainfluenzavirus in people with wheezing disorders. (PDF)

S9 Fig Global prevalence of human coronavirus in people with wheezing disorders. (PDF)

S10 Fig Funnel plot for publication for human respiratory syncytial virus in people with wheezing disorders. (PDF)

S11 Fig Funnel plot for publication for human metapneumovirus in people with wheezing disorders. (PDF)

S12 Fig Funnel plot for publication for influenza in people with wheezing disorders. (PDF)

S13 Fig Funnel plot for publication for rhinovirus in people with wheezing disorders. (PDF)

S14 Fig Funnel plot for publication for human adenovirus in people with wheezing disorders. (PDF)

S15 Fig Funnel plot for publication for human bocavirus in people with wheezing disorders. (PDF)

S16 Fig Funnel plot for publication for human parainfluenzavirus in people with wheezing disorders. (PDF)

S17 Fig Funnel plot for publication for human coronavirus in people with wheezing disorders. (PDF)

S18 Fig Funnel plot for publication for enterovirus in people with wheezing disorders. (PDF)

S1 Table Preferred reporting items for systematic reviews and meta-analyses checklist. (PDF)

S2 Table Search strategy in medline (Pubmed). (PDF)

S3 Table Items for risk of bias assessment. (PDF)

S4 Table Main reasons of exclusion of eligible studies. (PDF)

S5 Table Individual characteristics of included studies. (PDF)

S6 Table Subgroup prevalence of respiratory viral infections in people with wheezing. (PDF)

S7 Table Univariable and multivariable metaregression analysis on the prevalence of respiratory viruses in people with wheezing disorders. (PDF)

## References

[1] Alvarez-Alvarez I, Niu H, Guillen-Grima F, Aguinaga-Ontoso I. Meta-analysis of prevalence of wheezing and recurrent wheezing in infants. Allergol Immunopathol (Madr). 2018;46: 210–217. 10.1016/j.aller.2016.08.011 27865539

[2] Backman K, Ollikainen H, Piippo-Savolainen E, Nuolivirta K, Korppi M. Asthma and lung function in adulthood after a viral wheezing episode in early childhood. Clin Exp Allergy. 2018;48: 138–146. 10.1111/cea.13062 29143374

[3] Reijonen TM, Kotaniemi-Syrjänen A, Korhonen K, Korppi M. Predictors of asthma three years after hospital admission for wheezing in infancy. Pediatrics. 2000;106: 1406–1412. 10.1542/peds.106.6.1406 11099596

[4] Sigurs N, Bjarnason R, Sigurbergsson F, Kjellman B. Respiratory Syncytial Virus Bronchiolitis in Infancy Is an Important Risk Factor for Asthma and Allergy at Age 7. American Journal of Respiratory and Critical Care Medicine. 2000;161: 1501–1507. 10.1164/ajrccm.161.5.9906076 10806145

[5] Schauer U, Hoffjan S, Bittscheidt J, Köchling A, Hemmis S, Bongartz S, et al RSV bronchiolitis and risk of wheeze and allergic sensitisation in the first year of life. Eur Respir J. 2002;20: 1277–1283. 10.1183/09031936.02.00019902 12449185

[6] Fjaerli H-O, Farstad T, Bratlid D. Hospitalisations for respiratory syncytial virus bronchiolitis in Akershus, Norway, 1993–2000: a population-based retrospective study. BMC Pediatr. 2004;4: 25 10.1186/1471-2431-4-25 15606912 PMC544884

[7] Inoue Y, Shimojo N. Epidemiology of virus-induced wheezing/asthma in children. Front Microbiol. 2013;4 10.3389/fmicb.2013.00391 24379810 PMC3863784

[8] Martinez FD. Development of Wheezing Disorders and Asthma in Preschool Children. Pediatrics. 2002;109: 362–367. 11826251

[9] Johnston SL. The role of viral and atypical bacterial pathogens in asthma pathogenesis. Pediatr Pulmonol Suppl. 1999;18: 141–143. 10093125

[10] Robison RG, Singh AM. Chapter 11: the infant and toddler with wheezing. Allergy Asthma Proc. 2012;33 Suppl 1: 36–38. 10.2500/aap.2012.33.3543 22794684

[11] Pattemore PK, Johnston SL, Bardin PG. Viruses as precipitants of asthma symptoms. I. Epidemiology. Clinical & Experimental Allergy. 1992;22: 325–336. 10.1111/j.1365-2222.1992.tb03094.x 1586873 PMC7162032

[12] Gern JE, Busse WW. The role of viral infections in the natural history of asthma. Journal of Allergy and Clinical Immunology. 2000;106: 201–212. 10.1067/mai.2000.108604 10932062

[13] Savolainen C, Blomqvist S, Mulders MN, Hovi T. Genetic clustering of all 102 human rhinovirus prototype strains: serotype 87 is close to human enterovirus 70. J Gen Virol. 2002;83: 333–340. 10.1099/0022-1317-83-2-333 11807226

[14] Ginocchio CC. Detection of respiratory viruses using non-molecular based methods. J Clin Virol. 2007;40 Suppl 1: S11–14. 10.1016/S1386-6532(07)70004-5 18162248 PMC7130009

[15] Tuffaha A, Gern JE, Lemanske RF. The role of respiratory viruses in acute and chronic asthma. Clin Chest Med. 2000;21: 289–300. 10.1016/s0272-5231(05)70267-7 10907589 PMC7115729

[16] Gern JE. The ABCs of Rhinoviruses, Wheezing, and Asthma. Journal of Virology. 2010;84: 7418–7426. 10.1128/JVI.02290-09 20375160 PMC2897627

[17] Stenberg-Hammar K, Hedlin G, Söderhäll C. Rhinovirus and preschool wheeze. Pediatric Allergy and Immunology. 2017;28: 513–520. 10.1111/pai.12740 28599066

[18] Allander T, Tammi MT, Eriksson M, Bjerkner A, Tiveljung-Lindell A, Andersson B. Cloning of a human parvovirus by molecular screening of respiratory tract samples. Proc Natl Acad Sci U S A. 2005;102: 12891–12896. 10.1073/pnas.0504666102 16118271 PMC1200281

[19] van der Hoek L, Pyrc K, Jebbink MF, Vermeulen-Oost W, Berkhout RJM, Wolthers KC, et al Identification of a new human coronavirus. Nat Med. 2004;10: 368–373. 10.1038/nm1024 15034574 PMC7095789

[20] Noor A, Krilov LR. Respiratory syncytial virus vaccine: where are we now and what comes next? Expert Opin Biol Ther. 2018; 1–10. 10.1080/14712598.2018.1544239 30426788

[21] Moher D, Liberati A, Tetzlaff J, Altman DG. Preferred Reporting Items for Systematic Reviews and Meta-Analyses: The PRISMA Statement. PLoS Med. 2009;6 10.1371/journal.pmed.1000097 21603045 PMC3090117

[22] Ouzzani M, Hammady H, Fedorowicz Z, Elmagarmid A. Rayyan—a web and mobile app for systematic reviews. Syst Rev. 2016;5 10.1186/s13643-016-0384-4 27919275 PMC5139140

[23] Hoy D, Brooks P, Woolf A, Blyth F, March L, Bain C, et al Assessing risk of bias in prevalence studies: modification of an existing tool and evidence of interrater agreement. J Clin Epidemiol. 2012;65: 934–939. 10.1016/j.jclinepi.2011.11.014 22742910

[24] Viera AJ, Garrett JM. Understanding interobserver agreement: the kappa statistic. Fam Med. 2005;37: 360–363. 15883903

[25] Schwarzer G. meta: An R package for meta-analysis. R News. 2007;7: 40–5.

[26] R Core Team. R: A language and environment for statistical computing. R Foundation for Statistical Computing, Vienna, Austria 2017 [cited 28 Sep 2018]. Available: https://www.r-project.org/.

[27] Barendregt JJ, Doi SA, Lee YY, Norman RE, Vos T. Meta-analysis of prevalence. J Epidemiol Community Health. 2013;67: 974–978. 10.1136/jech-2013-203104 23963506

[28] Egger M, Davey Smith G, Schneider M, Minder C. Bias in meta-analysis detected by a simple, graphical test. BMJ. 1997;315: 629–634. 10.1136/bmj.315.7109.629 9310563 PMC2127453

[29] Cochran WG. The Combination of Estimates from Different Experiments. Biometrics. 1954;10: 101–129. 10.2307/3001666

[30] Higgins JPT, Thompson SG. Quantifying heterogeneity in a meta-analysis. Stat Med. 2002;21: 1539–1558. 10.1002/sim.1186 12111919

[31] Allander T, Jartti T, Gupta S, Niesters HGM, Lehtinen P, Üsterback R, et al Human Bocavirus and Acute Wheezing in Children. Clin Infect Dis. 2007;44: 904–910. 10.1086/512196 17342639 PMC7107819

[32] Bedolla-Barajas M, Montero H, Morales-Romero J, Landa-Cardeña A, Díaz J, Delgado-Figueroa N, et al Prevalence of respiratory viruses in wheezing children not older than 24 months of age. Gac Med Mex. 2017;153: 329–334. 28763071

[33] Bosis S, Esposito S, Niesters HGM, Zuccotti GV, Marseglia G, Lanari M, et al Role of respiratory pathogens in infants hospitalized for a first episode of wheezing and their impact on recurrences. Clin Microbiol Infect. 2008;14: 677–684. 10.1111/j.1469-0691.2008.02016.x 18558940 PMC7130007

[34] Camara AA, Silva JM, Ferriani VPL, Tobias KRC, Macedo IS, Padovani MA, et al Risk factors for wheezing in a subtropical environment: role of respiratory viruses and allergen sensitization. J Allergy Clin Immunol. 2004;113: 551–557. 10.1016/j.jaci.2003.11.027 15007360 PMC7127801

[35] Chung J-Y, Han TH, Kim SW, Kim CK, Hwang E-S. Detection of viruses identified recently in children with acute wheezing. J Med Virol. 2007;79: 1238–1243. 10.1002/jmv.20926 17597481 PMC7166823

[36] Cox DW, Bizzintino J, Ferrari G, Khoo SK, Zhang G, Whelan S, et al Human rhinovirus species C infection in young children with acute wheeze is associated with increased acute respiratory hospital admissions. American journal of respiratory and critical care medicine. 2013;188: 1358–64. 10.1164/rccm.201303-0498OC 23992536 PMC5447292

[37] de Winter JJH, Bont L, Wilbrink B, van der Ent CK, Smit HA, Houben ML. Rhinovirus wheezing illness in infancy is associated with medically attended third year wheezing in low risk infants: results of a healthy birth cohort study. Immun Inflamm Dis. 2015;3: 398–405. 10.1002/iid3.77 26734461 PMC4693725

[38] Deng Y, Liu E-M, Zhao X-D, Ding Y, Li Q-B, Luo Z-X, et al [Clinical characteristics of 12 persistently wheezing children with human bocavirus infection]. Zhonghua Er Ke Za Zhi. 2007;45: 732–735. 18211753

[39] Fuenzalida L, Fabrega J, Blanco S, Martinez M del M, Prat C, Pérez M, et al Usefulness of two new methods for diagnosing metapneumovirus infections in children. Clinical Microbiology and Infection. 2010;16: 1663–1668. 10.1111/j.1469-0691.2010.03192.x 20156218

[40] Fujitsuka A, Tsukagoshi H, Arakawa M, Goto-Sugai K, Ryo A, Okayama Y, et al A molecular epidemiological study of respiratory viruses detected in Japanese children with acute wheezing illness. BMC Infect Dis. 2011;11: 168 10.1186/1471-2334-11-168 21663657 PMC3123215

[41] García-García ML, Calvo C, Falcón A, Pozo F, Pérez-Breña P, De Cea JM, et al Role of emerging respiratory viruses in children with severe acute wheezing. Pediatr Pulmonol. 2010;45: 585–591. 10.1002/ppul.21225 20503284 PMC7167793

[42] Halmo Hurdum S, Zhang G, Khoo SK, Bizzintino J, Franks KM, Lindsay K, et al Recurrent rhinovirus detections in children following a rhinovirus-induced wheezing exacerbation: A retrospective study. International journal of pediatrics and child health. 2015;3: 10–18. 10.12974/2311-8687.2015.03.01.2 28018912 PMC5181795

[43] Hançerli-Törün S, Özçeker D, Uysalol M, Tamay Z, Şık G, Somer A, et al Predictive factor for first wheezing episode. Turk J Pediatr. 2015;57: 367–373. 27186699

[44] Jartti T, Lehtinen P, Vuorinen T, Österback R, van den Hoogen B, Osterhaus ADME, et al Respiratory Picornaviruses and Respiratory Syncytial Virus as Causative Agents of Acute Expiratory Wheezing in Children. Emerg Infect Dis. 2004;10: 1095–1101. 10.3201/eid1006.030629 15207063 PMC3323183

[45] Jartti T, van den Hoogen B, Garofalo RP, Osterhaus AD, Ruuskanen O. Metapneumovirus and acute wheezing in children. The Lancet. 2002;360: 1393–1394. 10.1016/S0140-6736(02)11391-2 12423987 PMC7119306

[46] Korppi M, Kotaniemi-Syrjänen A, Waris M, Vainionpää R, Reijonen TM. Rhinovirus-Associated Wheezing in Infancy: Comparison With Respiratory Syncytial Virus Bronchiolitis. The Pediatric Infectious Disease Journal. 2004;23: 995–999. 10.1097/01.inf.0000143642.72480.53 15545853

[47] Kotaniemi-Syrjänen A, Vainionpää R, Reijonen TM, Waris M, Korhonen K, Korppi M. Rhinovirus-induced wheezing in infancy—the first sign of childhood asthma? J Allergy Clin Immunol. 2003;111: 66–71. 10.1067/mai.2003.33 12532098 PMC7112360

[48] Lethbridge R, Prastanti F, Robertson C, Oo S, Khoo S-K, Le Souëf PN, et al Prospective Assessment of Rhinovirus Symptoms and Species Recurrence in Children With and Without an Acute Wheezing Exacerbation. Viral Immunol. 2018;31: 299–305. 10.1089/vim.2017.0152 29446705

[49] Moattari A, Aleyasin S, Arabpour M, Sadeghi S. Prevalence of human Metapneumovirus (hMPV) in children with wheezing in Shiraz-Iran. Iran J Allergy Asthma Immunol. 2010;9: 250–254. doi: 09.04/ijaai.251254 21131706

[50] Mummidi PS, Tripathy R, Dwibedi B, Mahapatra A, Baraha S. Viral aetiology of wheezing in children under five. Indian J Med Res. 2017;145: 189–193. 10.4103/ijmr.IJMR_840_15 28639594 PMC5501050

[51] Ong BH, Gao Q, Phoon MC, Chow VT, Tan WC, Van Bever HP. Identification of human metapneumovirus and Chlamydophila pneumoniae in children with asthma and wheeze in Singapore. Singapore medical journal. 2007;48: 291–3. 17384874

[52] Piotrowska Z, Vázquez M, Shapiro ED, Weibel C, Ferguson D, Landry ML, et al Rhinoviruses are a major cause of wheezing and hospitalization in children less than 2 years of age. The Pediatric infectious disease journal. 2009;28: 25 10.1097/INF.0b013e3181861da0 19057454 PMC4639321

[53] Sackesen C, Pinar A, Sekerel BE, Akyon Y, Saraclar Y. Use of polymerase chain reaction for detection of adenovirus in children with or without wheezing. The Turkish journal of pediatrics. 2005;47: 227–31. 16250306

[54] Shen J, Zhu Q, Zeng M, Yu H. Detection and genome analysis of human bocavirus 1–4 from hospitalized children with acute lower respiratory tract infection and symptoms of wheezing in Shanghai. Int J Mol Med. 2013;32: 1415–1420. 10.3892/ijmm.2013.1512 24085194

[55] Smuts HE, Workman LJ, Zar HJ. Human rhinovirus infection in young African children with acute wheezing. BMC Infect Dis. 2011;11: 65 10.1186/1471-2334-11-65 21401965 PMC3065410

[56] Smuts H, Workman L, Zar HJ. Role of human metapneumovirus, human coronavirus NL63 and human bocavirus in infants and young children with acute wheezing. J Med Virol. 2008;80: 906–912. 10.1002/jmv.21135 18360904 PMC7166566

[57] Stenberg-Hammar K, Niespodziana K, Söderhäll C, James A, Cabauatan CR, Konradsen JR, et al Rhinovirus-specific antibody responses in preschool children with acute wheeze reflect severity of respiratory symptoms. Allergy. 2016;71: 1728–1735. 10.1111/all.12991 27444786

[58] Sun H, Sun Q, Jiang W, Chen Z, Huang L, Wang M, et al Prevalence of rhinovirus in wheezing children: a comparison with respiratory syncytial virus wheezing. Braz J Infect Dis. 2016 10.1016/j.bjid.2015.12.005 26859065 PMC9427575

[59] Takeyama A, Hashimoto K, Sato M, Sato T, Tomita Y, Maeda R, et al Clinical and epidemiologic factors related to subsequent wheezing after virus-induced lower respiratory tract infections in hospitalized pediatric patients younger than 3 years. Eur J Pediatr. 2014;173: 959–966. 10.1007/s00431-014-2277-7 24535712

[60] Teeratakulpisarn J, Pientong C, Ekalaksananan T, Ruangsiripiyakul H, Uppala R. Rhinovirus infection in children hospitalized with acute bronchiolitis and its impact on subsequent wheezing or asthma: a comparison of etiologies. Asian Pac J Allergy Immunol. 2014;32: 226–234. 10.12932/AP0417.32.3.2014 25268340

[61] Turunen R, Koistinen A, Vuorinen T, Arku B, Söderlund-Venermo M, Ruuskanen O, et al The first wheezing episode: respiratory virus etiology, atopic characteristics, and illness severity. Pediatr Allergy Immunol. 2014;25: 796–803. 10.1111/pai.12318 25444257 PMC7167827

[62] van der Schee MP, Hashimoto S, Schuurman AC, van Driel JSR, Adriaens N, van Amelsfoort RM, et al Altered exhaled biomarker profiles in children during and after rhinovirus-induced wheeze. Eur Respir J. 2015;45: 440–448. 10.1183/09031936.00044414 25323245

[63] van der Zalm MM, Uiterwaal CSPM, Wilbrink B, Koopman M, Verheij TJM, van der Ent CK. The influence of neonatal lung function on rhinovirus-associated wheeze. Am J Respir Crit Care Med. 2011;183: 262–267. 10.1164/rccm.200905-0716OC 20802166

[64] Message SD, Johnston SL. Viruses in asthma. Br Med Bull. 2002;61: 29–43. 10.1093/bmb/61.1.29 11997297 PMC7110059

[65] Zheng X-Y, Xu Y-J, Guan W-J, Lin L-F. Regional, age and respiratory-secretion-specific prevalence of respiratory viruses associated with asthma exacerbation: a literature review. Arch Virol. 2018;163: 845–853. 10.1007/s00705-017-3700-y 29327237 PMC7087223

[66] Liu L, Pan Y, Zhu Y, Song Y, Su X, Yang L, et al Association between rhinovirus wheezing illness and the development of childhood asthma: a meta-analysis. BMJ Open. 2017;7: e013034 10.1136/bmjopen-2016-013034 28373249 PMC5387933

[67] Rylander E, Eriksson M, Pershagen G, Nordvall L, Ehrnsr A, Ziegler T. Wheezing bronchitis in children. Incidence, viral infections, and other risk factors in a defined population. Pediatric allergy and immunology. 1996;7: 6–11. 10.1111/j.1399-3038.1996.tb00099.x 8792378

[68] McIntosh K, Ellis EF, Hoffman LS, Lybass TG, Eller JJ, Fulginiti VA. The association of viral and bacterial respiratory infections with exacerbations of wheezing in young asthmatic children. The Journal of Pediatrics. 1973;82: 578–590. 10.1016/s0022-3476(73)80582-7 4349062 PMC7130678

[69] JohnstonSL. Influence of viral and bacterial respiratory infections on exacerbations and symptom severity in childhood asthma. Pediatr Pulmonol Suppl. 1997;16: 88–89. 10.1002/ppul.1950230851 9443219

[70] Johnston SL, Pattemore PK, Sanderson G, Smith S, Campbell MJ, Josephs LK, et al The relationship between upper respiratory infections and hospital admissions for asthma: a time-trend analysis. Am J Respir Crit Care Med. 1996;154: 654–660. 10.1164/ajrccm.154.3.8810601 8810601

[71] American Academy of Pediatrics Subcommittee on Diagnosis and Management of Bronchiolitis. Diagnosis and management of bronchiolitis. Pediatrics. 2006;118: 1774–1793. 10.1542/peds.2006-2223 17015575

[72] Faux CE, Arden KE, Lambert SB, Nissen MD, Nolan TM, Chang AB, et al Usefulness of published PCR primers in detecting human rhinovirus infection. Emerging Infect Dis. 2011;17: 296–298. 10.3201/eid1702.101123 21291610 PMC3204776

[73] Sakthivel SK, Whitaker B, Lu X, Oliveira DBL, Stockman LJ, Kamili S, et al Comparison of fast-track diagnostics respiratory pathogens multiplex real-time RT-PCR assay with in-house singleplex assays for comprehensive detection of human respiratory viruses. Journal of Virological Methods. 2012;185: 259–266. 10.1016/j.jviromet.2012.07.010 22796035 PMC7119496

[74] Broccolo F, Falcone V, Esposito S, Toniolo A. Human bocaviruses: Possible etiologic role in respiratory infection. J Clin Virol. 2015;72: 75–81. 10.1016/j.jcv.2015.09.008 26441386

