# Supplementary figures and images for: Prevalence of respiratory viruses using polymerase chain reaction in children with wheezing, a systematic review and meta–analysis
Source: PLoS One. 2020 Dec 14;15(12):e0243735. doi: 10.1371/journal.pone.0243735 (PMC7735590; doi:10.1371/journal.pone.0243735)

S1 Fig. Global prevalence of Rhinovirus in people with wheezing disorders

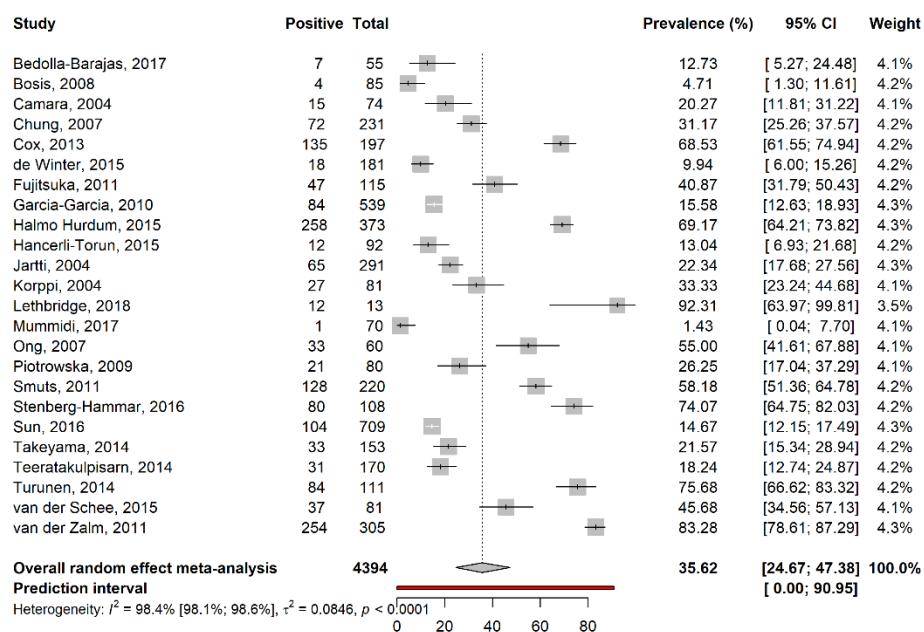

Supplement: S1 Fig — (PDF) [file pone.0243735.s001.pdf]

S3 Fig. Global prevalence of Human Bocavirus in people with wheezing disorders

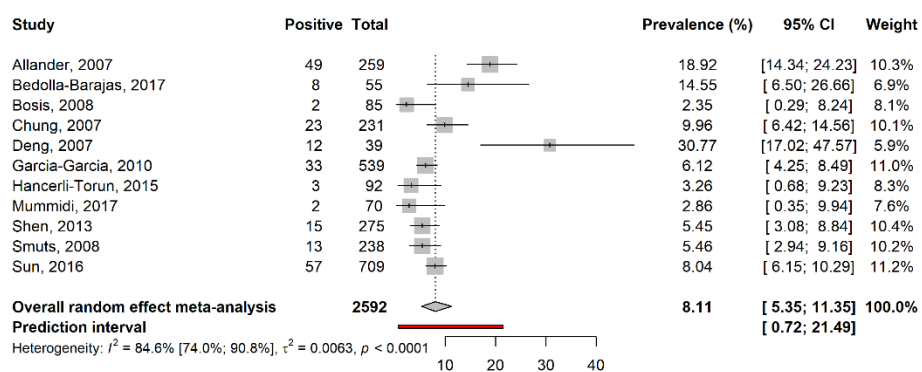

Supplement: S3 Fig — (PDF) [file pone.0243735.s003.pdf]

S4 Fig. Global prevalence of Human Adenovirus in people with wheezing disorders

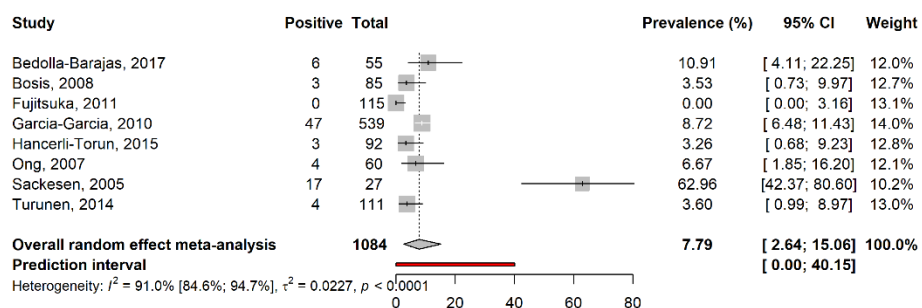

Supplement: S4 Fig — (PDF) [file pone.0243735.s004.pdf]

S5 Fig. Global prevalence of Influenza in people with wheezing disorders

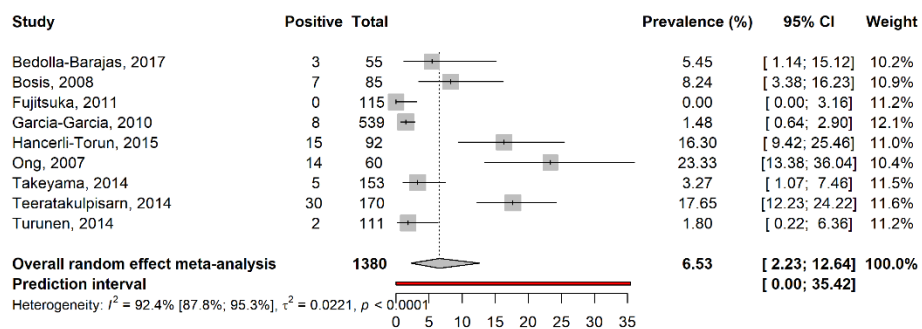

Supplement: S5 Fig — (PDF) [file pone.0243735.s005.pdf]

S6 Fig. Global prevalence of Human Metapneumovirus in people with wheezing disorders

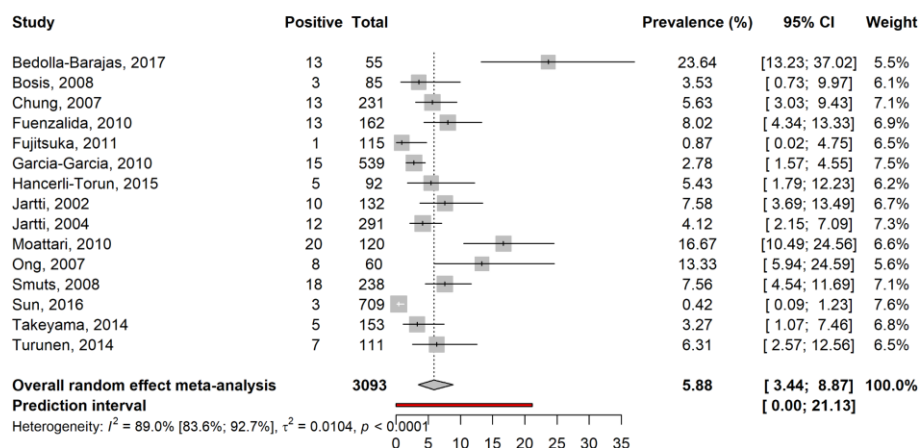

Supplement: S6 Fig — (PDF) [file pone.0243735.s006.pdf]

S7 Fig. Global prevalence of Enterovirus in people with wheezing disorders

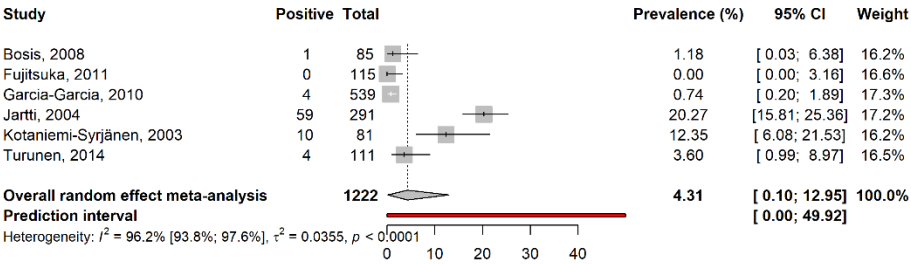

Supplement: S7 Fig — (PDF) [file pone.0243735.s007.pdf]

S8 Fig. Global prevalence of Human Parainfluenza Virus in people with wheezing disorders

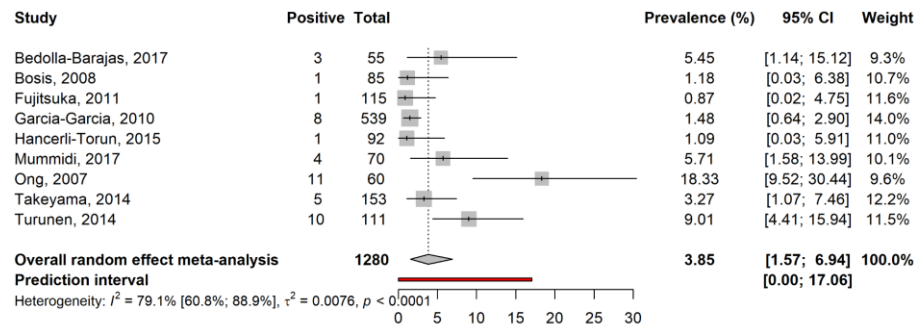

Supplement: S8 Fig — (PDF) [file pone.0243735.s008.pdf]

S9 Fig. Global prevalence of Human Coronavirus in people with wheezing disorders

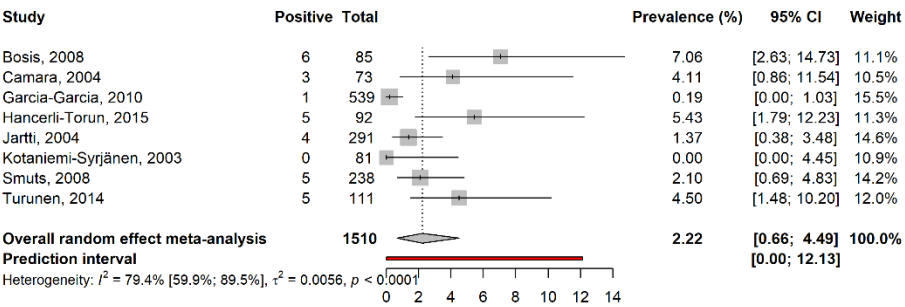

Supplement: S9 Fig — (PDF) [file pone.0243735.s009.pdf]

S10 Fig. Funnel plot for publication for HRSV in people with wheezing disorders

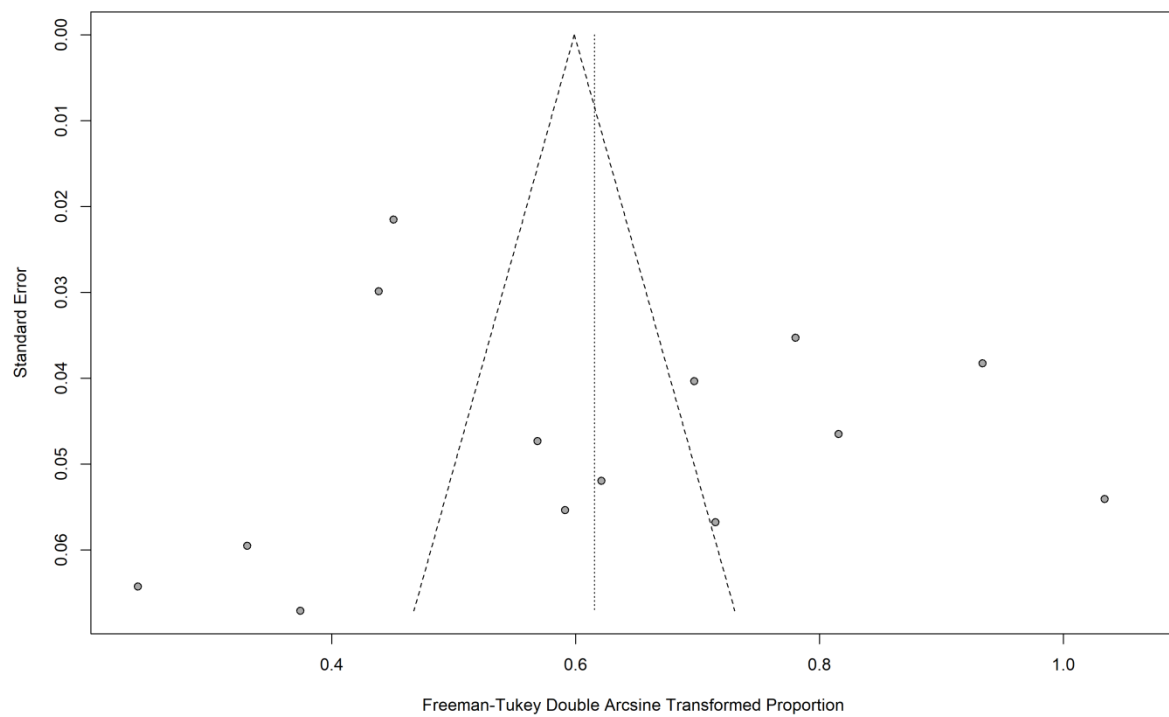

Supplement: S10 Fig — (PDF) [file pone.0243735.s010.pdf]

S11 Fig. Funnel plot for publication for HMPV in people with wheezing disorders

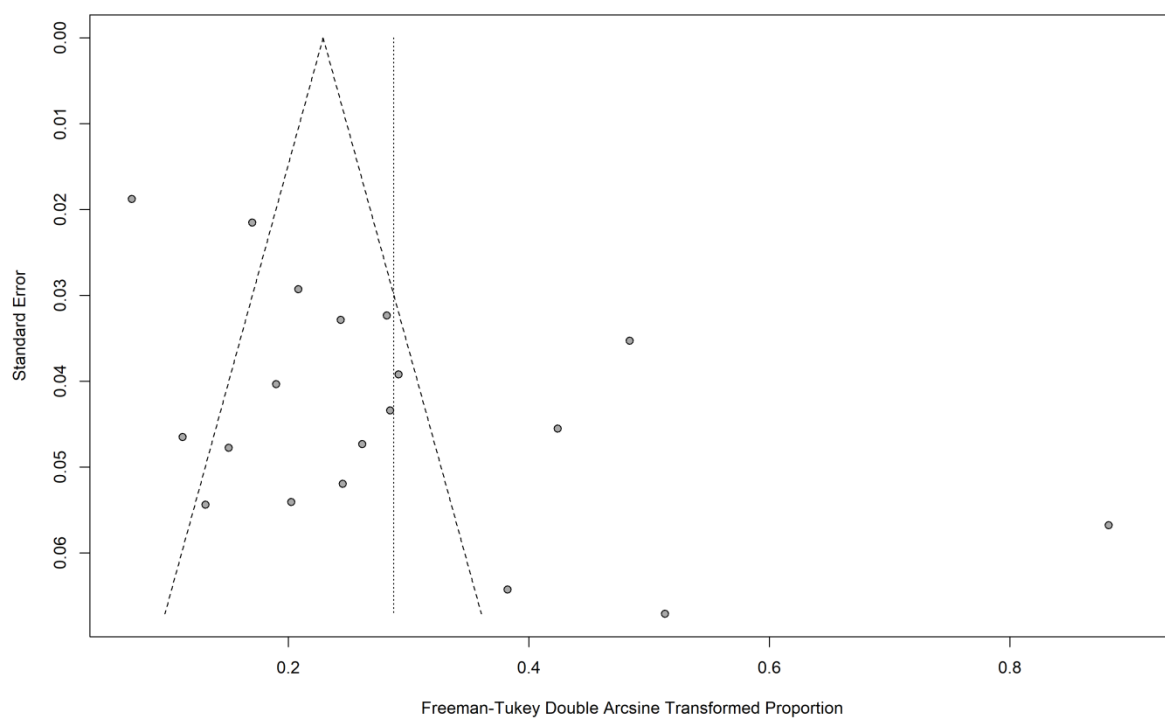

Supplement: S11 Fig — (PDF) [file pone.0243735.s011.pdf]

S12 Fig. Funnel plot for publication for Influenza in people with wheezing disorders

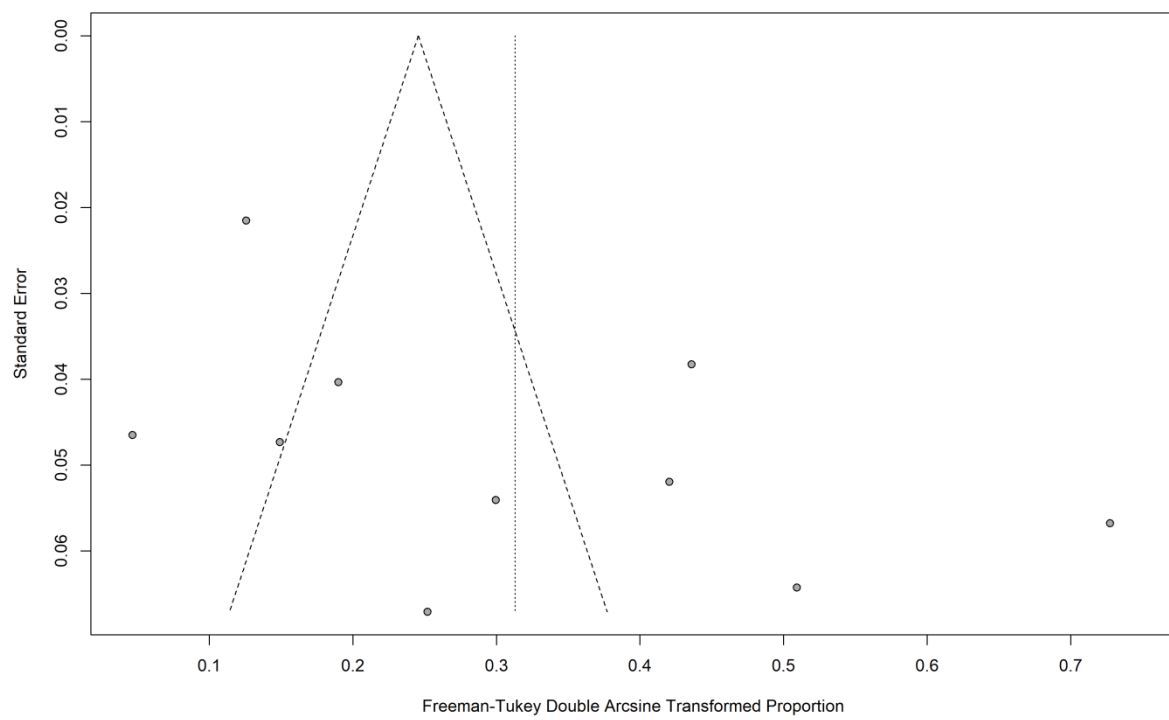

Supplement: S12 Fig — (PDF) [file pone.0243735.s012.pdf]

S13 Fig. Funnel plot for publication for RV in people with wheezing disorders

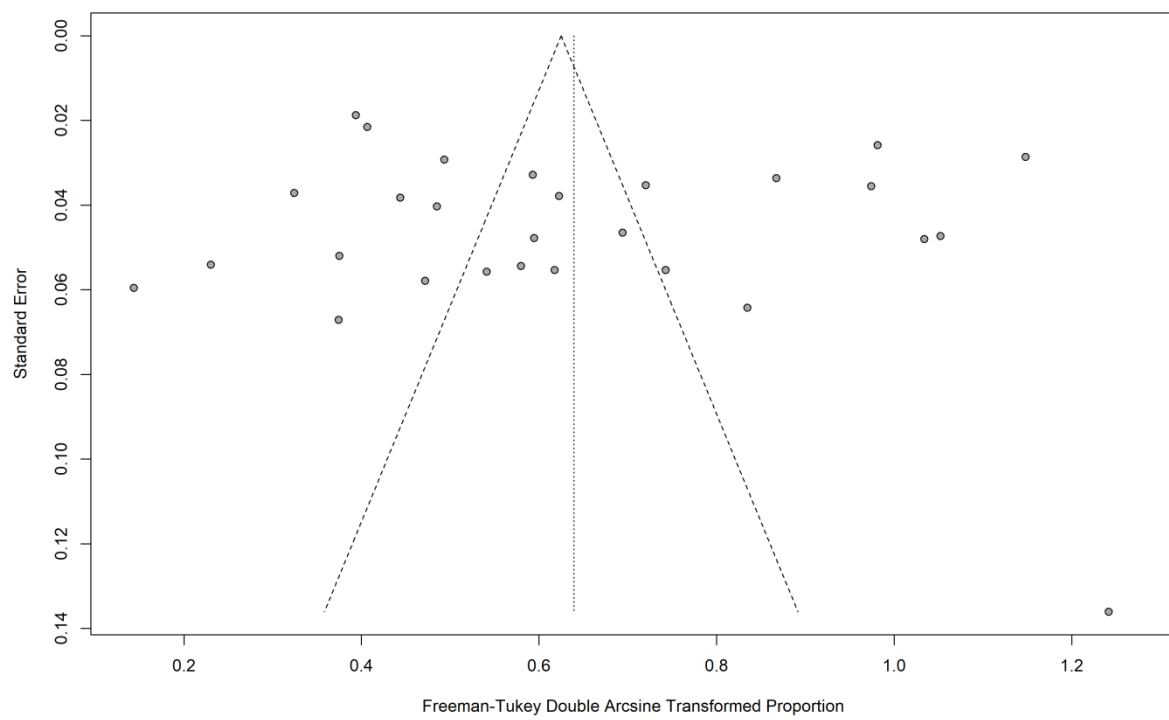

Supplement: S13 Fig — (PDF) [file pone.0243735.s013.pdf]

S14 Fig. Funnel plot for publication for HAdV in people with wheezing disorders

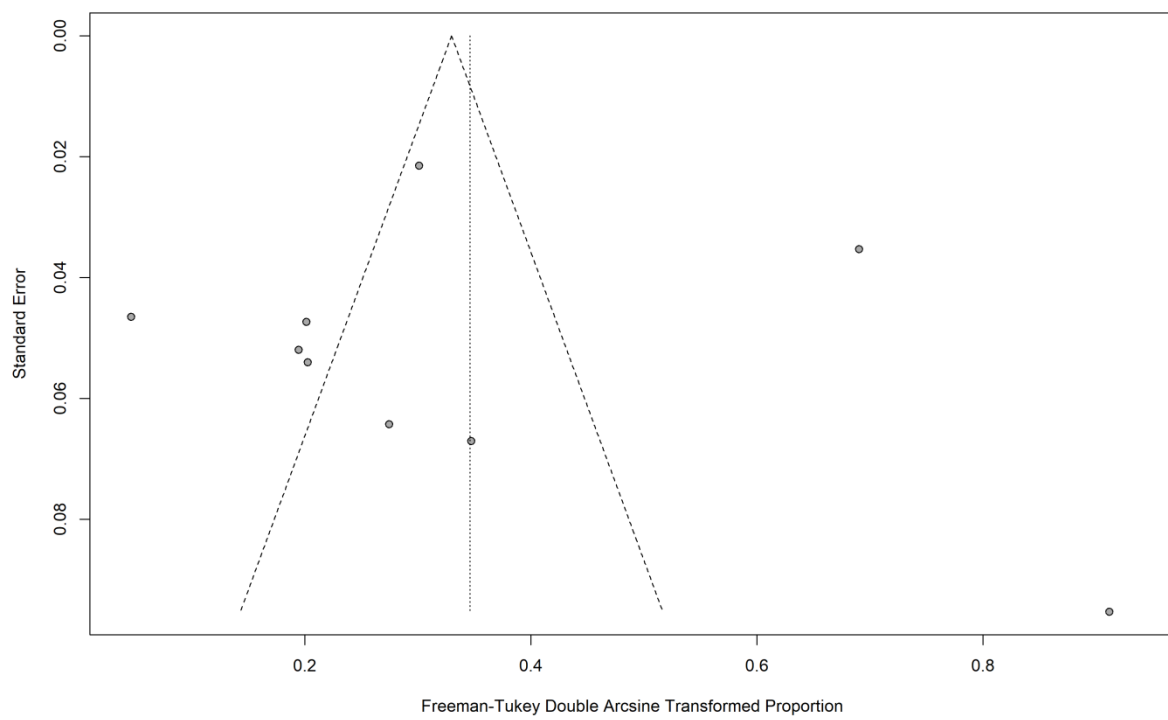

Supplement: S14 Fig — (PDF) [file pone.0243735.s014.pdf]

S15 Fig. Funnel plot for publication for HBoV in people with wheezing disorders

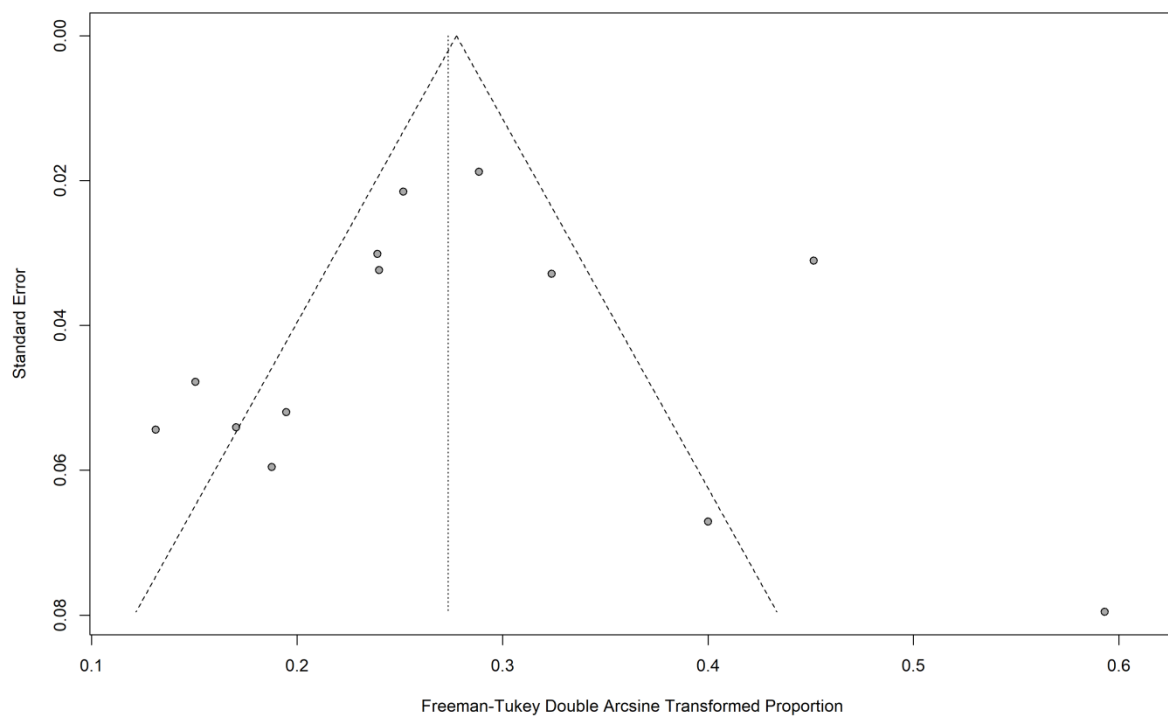

Supplement: S15 Fig — (PDF) [file pone.0243735.s015.pdf]

S16 Fig. Funnel plot for publication for HPIV in people with wheezing disorders

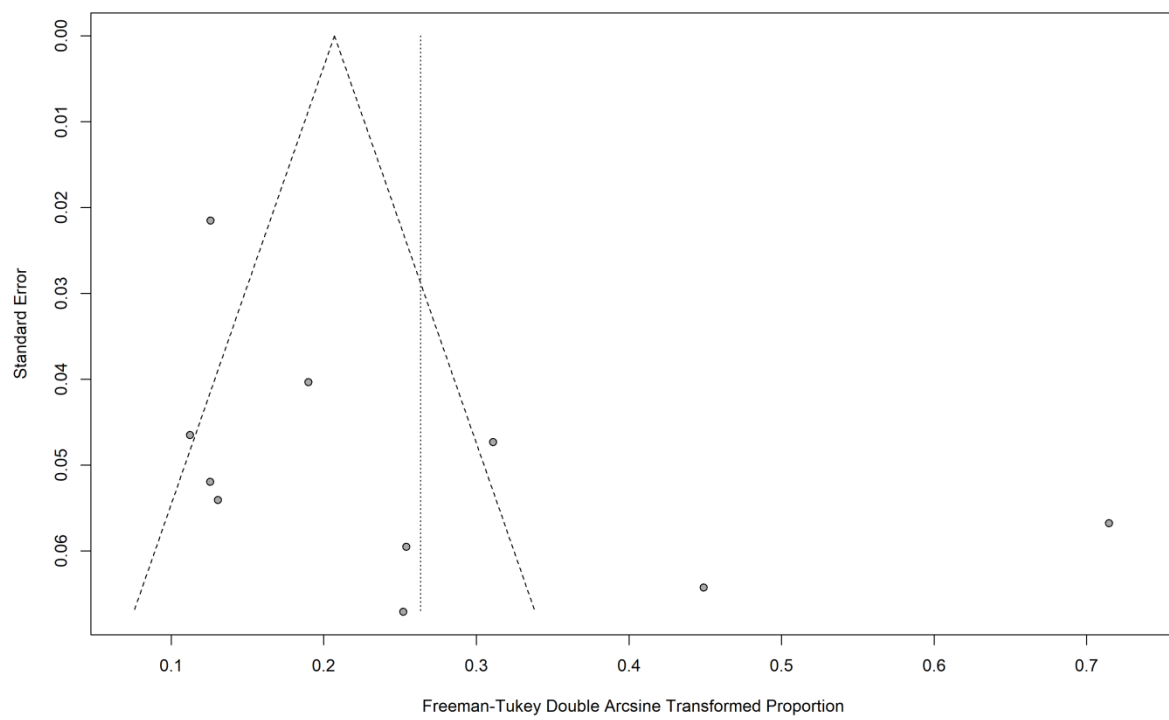

Supplement: S16 Fig — (PDF) [file pone.0243735.s016.pdf]

S17 Fig. Funnel plot for publication for HCoV in people with wheezing disorders

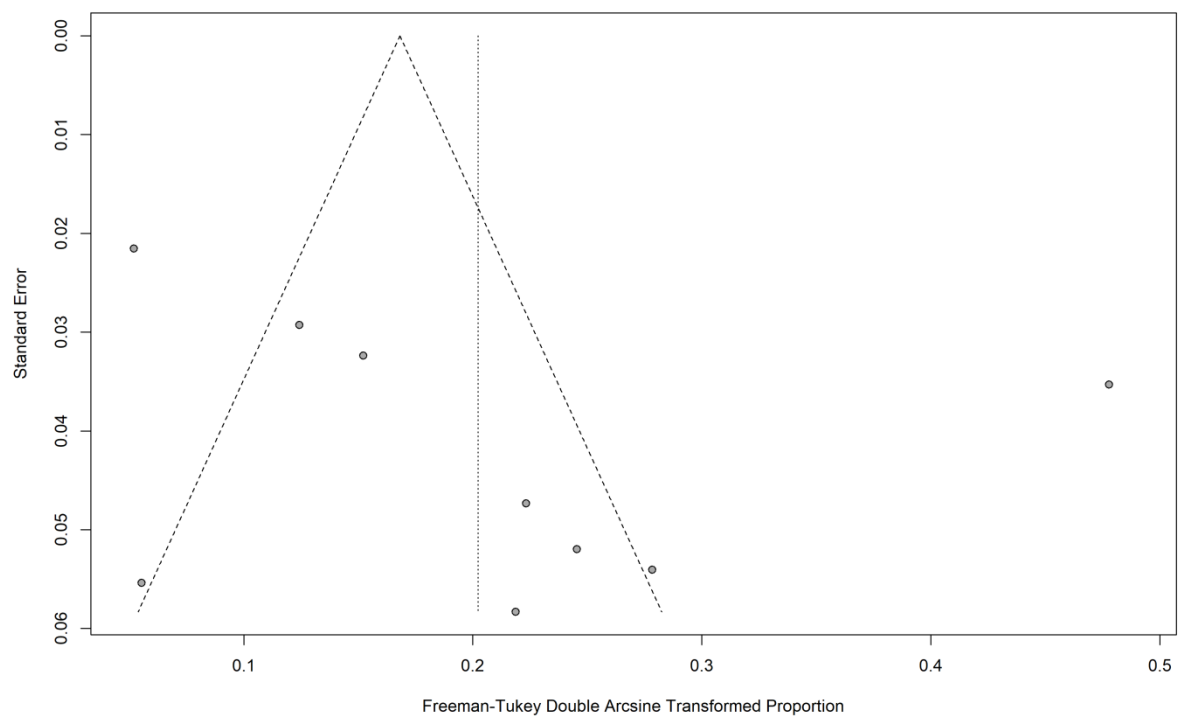

Supplement: S17 Fig — (PDF) [file pone.0243735.s017.pdf]

S18 Fig. Funnel plot for publication for EV in people with wheezing disorders

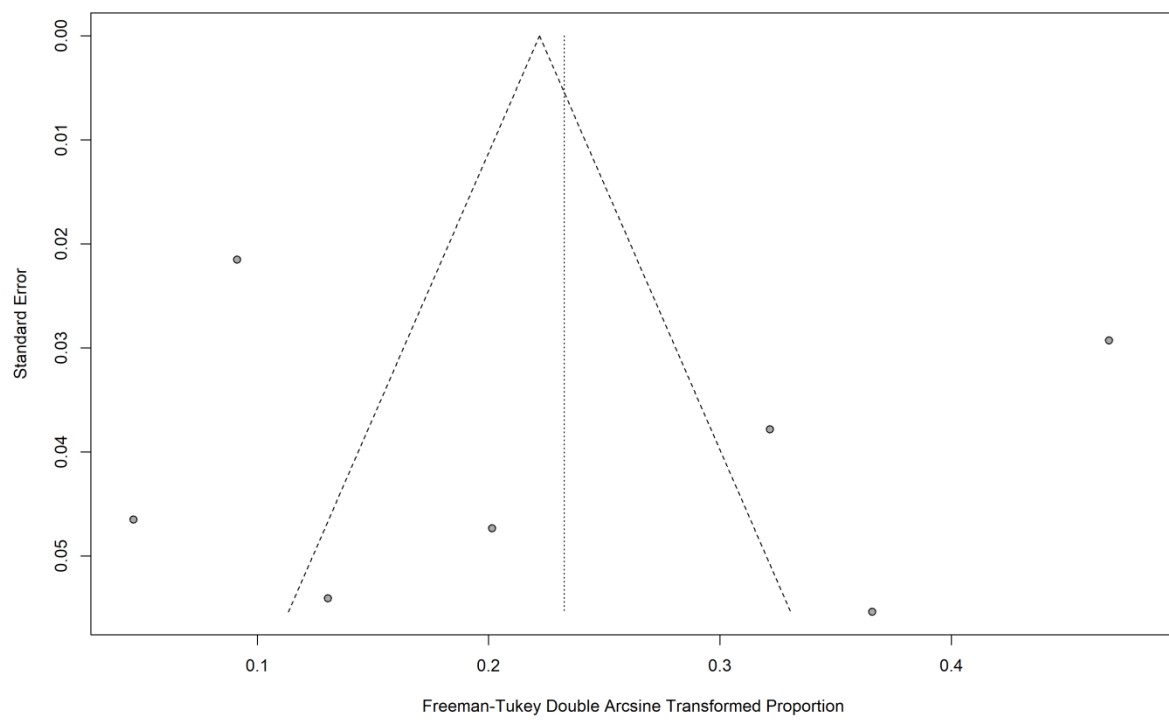

Supplement: S18 Fig — (PDF) [file pone.0243735.s018.pdf]
