# Supplementary material for: Prevalence of respiratory viruses using polymerase chain reaction in children with wheezing, a systematic review and meta–analysis
Source: PLoS One. 2020 Dec 14;15(12):e0243735. doi: 10.1371/journal.pone.0243735 (PMC7735590; doi:10.1371/journal.pone.0243735)

S2 Fig. Global prevalence of Human Respiratory Syncytial Virus in people with wheezing disorders

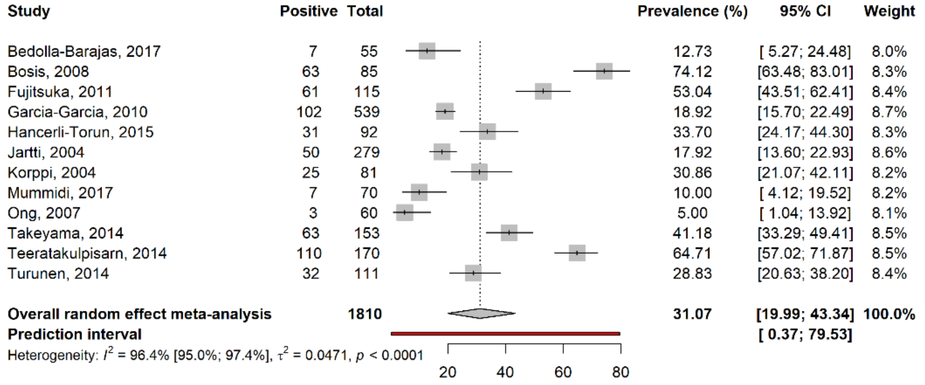

Supplement: S2 Fig — (PDF) [file pone.0243735.s002.pdf]
