# Supplementary material for: Prevalence of respiratory viruses using polymerase chain reaction in children with wheezing, a systematic review and meta–analysis
Source: PLoS One. 2020 Dec 14;15(12):e0243735. doi: 10.1371/journal.pone.0243735 (PMC7735590; doi:10.1371/journal.pone.0243735)
Supplement: S2 Table — (PDF) [file pone.0243735.s020.pdf]

S2 Table. Search strategy in Medline (Pubmed)

Search	Virus
#1	"HRSV" OR "RSV" OR "human respiratory syncytial virus"OR "respiratory syncytial virus" OR "HRSV-A" OR "HRSV-B" OR "HMPV" OR "MPV" OR "human metapneumovirus"OR "metapneumovirus" OR "HMPV-A" OR "HMPV-B" OR "HAdV" OR "AdV" OR "Adenovirus" OR "Adenovirus Infections, Human" OR "Human adenovirus" OR "HADV-A" OR "HADV-B" OR "HADV-C" OR "HADV-D" OR "HADV-E" OR "HADV-F" OR "HADV-G" OR "HBoV" OR "BoV" OR "Bocavirus" OR "Bocavirus Infections, Human" OR "Human Bocavirus" OR "HCoV" OR "CoV" OR "Coronavirus" OR "Coronavirus Infections, Human" OR "Human Coronavirus" OR "229E" OR "OC43" OR "NL63" OR "HKU1" OR "HCoV-229E" OR "HCoV-OC43" OR "HCoV-NL63" OR "HCoV-HKU1" OR "HPIV" OR "PIV" OR "Parainfluenzavirus" OR "Parainfluenzavirus Infections, Human" OR "Human Parainfluenzavirus" OR "PIV-1" OR "PIV-2" OR "PIV-3" OR "PIV-4" OR "HPIV-1" OR "HPIV-2" OR "HPIV-3" OR "HPIV-4" OR "HEV" OR "EV" OR "Enterovirus" OR "Enterovirus Infections, Human" OR "Human Enterovirus" OR "HRV" OR "RV" OR "Rhinovirus" OR "Rhinoviruses" OR "Rhinovirus Infections, Human" OR "Human Rhinovirus" OR "RV-A" OR "RV-B" OR "RV-C" OR "Influenza" OR "Inf" OR "Influenza virus" OR "Influenza, Human" OR "Influenza-A virus" OR "Influenza-B virus" OR "Influenza-C virus"
#2	"wheezing" OR "recurrent wheezing" OR "first wheezing" OR "first wheezing episode" OR "subsequent wheezing" OR "wheezing illnesses" OR "wheeze" OR "persistent recurring wheezing" OR "subsequent recurrent wheezing" OR "wheezing episode" OR "late wheezing" OR "acute wheezing" OR "early wheezing" OR "acute expiratory wheezing" OR "wheezy bronchitis" OR "wheeze-associated RTI" OR "wheezing episodes"
#3	(#1 AND #2)
