# Supplementary material for: Prevalence of respiratory viruses using polymerase chain reaction in children with wheezing, a systematic review and meta–analysis
Source: PLoS One. 2020 Dec 14;15(12):e0243735. doi: 10.1371/journal.pone.0243735 (PMC7735590; doi:10.1371/journal.pone.0243735)
Supplement: S4 Table — (PDF) [file pone.0243735.s022.pdf]

S4 Table. Main reasons of exclusion of eligible studies

N°	Name, Year	Title	Reason of exclusion
1	Aakula, 2014	The influence of viral or bacterial coinfection on the clinical outcomes of rhinovirus-induced wheeze in hospitalised children.	Conference abstract
2	Alyasin, 2010	Human metapneumovirus in wheezy children admitted in Shiraz, Iran.	Conference abstract
3	Alyasin, 2011	Human-metapneumovirus in children admitted with wheeze.	Conference abstract
4	Alyasin, 2013	Co-infection of RSV and h-MPV in wheezy children admitted in Shiraz, Iran.	Conference abstract
5	Anderson, 2017	Assessment of wheezing frequency and viral etiology on childhood and adolescent asthma risk	No data on viral etiology searched
6	Annamalay, 2017	Rhinovirus C is associated with wheezing and rhinovirus A is associated with pneumonia in hospitalized children in Morocco	No wheezing participants recruited
7	Arden, 2010	Molecular characterization and distinguishing features of a novel human rhinovirus (HRV) C HRVC-QCE detected in children with fever cough and wheeze during 2003	No wheezing participants recruited
8	Backman, 2018	Asthma and lung function in adulthood after a viral wheezing episode in early childhood	No PCR was used
9	Balfour-Lynn, 1995	Diagnosing respiratory syncytial virus by nasal lavage	No data on viral etiology searched
10	Beigelman, 2014	Infection-induced wheezing in young children	Review
11	Berce, 2017	Viral infections in preschool children with wheezing.	Conference abstract
12	Blanken, 2013	Respiratory syncytial virus and recurrent wheeze	Only participants with comorbidities
13	Calvo, 2017	[Respiratory viral infections in a cohort of children during the first year of life and their role in the development of wheezing]	No wheezing participants recruited
14	Carroll, 2008	The Impact of Respiratory Viral Infection on Wheezing Illnesses and Asthma Exacerbations	Review
15	Castro-Rodriguez, 2001	Relation of two different subtypes of croup before age three to wheezing atopy and pulmonary function during childhood: a prospective study	No wheezing participants recruited
16	Chieochansin, 2008	Human bocavirus (HBoV) in Thailand: clinical manifestations in a hospitalized pediatric patient and molecular virus characterization	No wheezing participants recruited
17	Chung, 2002	RANTES may be predictive of later recurrent wheezing after respiratory syncytial virus bronchiolitis in infants	No data on viral etiology searched
18	Cifuentes, 2003	Risk factors for recurrent wheezing following acute bronchiolitis: a 12-month follow-up	No wheezing participants recruited
19	Coleman, 2015	Rhinovirus A and C wheezing illness in infancy and the development of asthma.	Conference abstract
20	Coverstone, 2018	Recurrent wheezing in children following human metapneumovirus infection	No wheezing participants recruited
21	Dabaniyasti, 2012	Investigation of respiratory viruses by molecular method in children with acute lower respiratory tract infection or wheezing.	Conference abstract
22	Dabaniyasti, 2016	An investigation into respiratory tract viruses in children with acute lower respiratory tract infection or wheezing	Complete text not found
23	Dawood, 2016	Incidence and characteristics of early childhood wheezing Dhaka Bangladesh 2004-2010	No wheezing participants recruited

24	De Alarcon, 2001	Detection of IgA and IgG but not IgE antibody to respiratory syncytial virus in nasal washes and sera from infants with wheezing	No continuous study period
25	Deerojanawong, 2013	Incidence of recurrent wheezing in under 5-year-old human bocavirus infection during one year follow-up	No wheezing participants recruited
26	Del Rosal, 2016	Recurrent wheezing and asthma after bocavirus bronchiolitis	No wheezing participants recruited
27	Deng, 2012	High Viral Load of Human Bocavirus Correlates with Duration of Wheezing in Children with Severe Lower Respiratory Tract Infection	No wheezing participants recruited
28	Duff, 1993	Risk factors for acute wheezing in infants and children: viruses passive smoke and IgE antibodies to inhalant allergens	No wheezing participants recruited
29	El-Gamal, 2011	Wheezing in infancy	Review
30	Eriksson, 2000	Wheezing following lower respiratory tract infections with respiratory syncytial virus and influenza A in infancy	No wheezing participants recruited
31	Ermers, 2007	IL-13 genetic polymorphism identifies children with late wheezing after respiratory syncytial virus infection	No wheezing participants recruited
32	Escobar, 2010	Recurrent wheezing in the third year of life among children born at 32 weeks' gestation or later: relationship to laboratory-confirmed medically attended infection with respiratory syncytial virus during the first year of life	No wheezing participants recruited
33	Escobar, 2013	Persistent recurring wheezing in the fifth year of life after laboratory-confirmed medically attended respiratory syncytial virus infection in infancy	No wheezing participants recruited
34	Farah, 2002	First-time wheezing in infants during respiratory syncytial virus season: chest radiograph findings	No data on viral etiology searched
35	Fjærli, 2005	Acute bronchiolitis in infancy as risk factor for wheezing and reduced pulmonary function by seven years in Akershus County Norway	No wheezing participants recruited
36	Freeman, 1966	Wheezing associated with respiratory tract infections in children. The role of specific infectious agents in allergic respiratory manifestations.	Review
37	Friedman, 2013	Viral etiology for acute wheezing episodes in children with high risk for asthama.	Conference abstract
38	Gern, 2002	Relationship of viral infections to wheezing illnesses and asthma	Review
39	Gonzalez-Uribe, 2017	Epidemiological transition of viruses associated with wheezing. The role of new virus.	Conference abstract
40	Gu, 2017	Elevated Serum Levels of Thymic Stromal Lymphopoietin in Wheezing Children Infected with Human Metapneumovirus	No wheezing participants recruited
41	Guilbert, 2011	Decreased Lung Function After Preschool Wheezing Rhinovirus Illnesses in Children At Risk to Develop Asthma	No data on viral etiology searched
42	Guilbert, 2011	Decreased lung function after preschool wheezing rhinovirus illnesses in children at risk to develop asthma.	No wheezing participants recruited
43	Halmo Hurdum, 2014	Following hospital presentation with a wheezing exacerbation, children with a parental history of asthma or allergies have increased recurrence of human rhinovirus (HRV).	Conference abstract
44	Heymann, 1995	Assessment of eosinophils viruses and IgE antibody in wheezing infants and children	No data on viral etiology searched
45	Heymann, 2004	Viral infections in relation to age atopy and season of admission among children hospitalized for wheezing	No data on viral etiology searched

46	Heymann, 2005	Role of viral infections, atopy and antiviral immunity in the etiology of wheezing exacerbations among children and young adults.	Review
47	Horn, 1979	Role of viruses and bacteria in acute wheezy bronchitis in childhood: a study of sputum	No wheezing participants recruited
48	Hürdum, 2013	Wheezing children with human rhinovirus species C have a history of respiratory viral infections.	Conference abstract
49	Hyvarinen, 2005	Teenage asthma after severe early childhood wheezing: an 11-year prospective follow-up	No data on viral etiology searched
50	Inoue, 2013	Epidemiology of virus-induced wheezing/asthma in children	Review
51	Jackson, 2008	Wheezing Rhinovirus Illnesses in Early Life Predict Asthma Development in High-Risk Children	No data on viral etiology searched
52	Jackson, 2010	Allergic sensitization is a risk factor for rhinovirus wheezing illnesses during early childhood.	Conference abstract
53	Jackson, 2012	Evidence for a Causal Relationship between Allergic Sensitization and Rhinovirus Wheezing in Early Life	No data on viral etiology searched
54	Jartti, 2006	Evaluation of the efficacy of prednisolone in early wheezing induced by rhinovirus or respiratory syncytial virus	No data on viral etiology searched
55	Jartti, 2009	Bronchiolitis: age and previous wheezing episodes are linked to viral etiology and atopic characteristics	duplicate
56	Jartti, 2009	The clinical importance of rhinovirus-associated early wheezing.	Comment
57	Jartti, 2010	Low serum 25-hydroxyvitamin D levels are associated with increased risk of viral coinfections in wheezing children	No continuous study period
58	Jartti, 2010	Allergic sensitization is associated with rhinovirus- but not other virus- induced wheezing in children	No data on viral etiology searched
59	Kato, 2015	Virus detection and cytokine profile in relation to age among acute exacerbations of childhood wheezing.	duplicate
60	Korppi, 2015	Virus-induced wheezing in infants aged 12-24 months and bronchiolitis in infants under 6 months are different clinical entities.	Comment
61	Kotaniemi-Syrjanen, 2002	Wheezing requiring hospitalization in early childhood: predictive factors for asthma in a six-year follow-up.	No PCR was used
62	Kotaniemi-Syrjanen, 2005	Respiratory syncytial virus infection in children hospitalized for wheezing: virus-specific studies from infancy to preschool years	duplicate
63	Kotaniemi-Syrjanen, 2007	Airway responsiveness: associated features in infants with recurrent respiratory symptoms	No data on viral etiology searched
64	Kotaniemi-Syrjänen, 2008	Wheezing due to rhinovirus infection in infancy: Bronchial hyperresponsiveness at school age	duplicate
65	Lazzaro, 2007	Respiratory syncytial virus infection and recurrent wheeze/asthma in children under five years: an epidemiological survey	No PCR was used
66	Lehtinen, 2006	Bacterial coinfections in children with viral wheezing	No data on viral etiology searched
67	Lehtinen, 2007	Prednisolone reduces recurrent wheezing after a first wheezing episode associated with rhinovirus infection or eczema	duplicate
68	Leino, 2018	Pulmonary function and bronchial reactivity 4 years after the first virus-induced wheezing	duplicate
69	Linsuwanon, 2009	Recurrent human rhinovirus infections in infants with refractory wheezing	Case report
70	Liu, 2016	Clinical characteristics and lung function in hospitalized infants with wheezing lower respiratory tract infections in China.	Conference abstract
71	Lopez Perez, 2009	Identification of influenza parainfluenza adenovirus and respiratory syncytial virus during rhinopharyngitis in a group of Mexican children with asthma and wheezing	No wheezing participants recruited

72	Lu, 2014	Molecular epidemiology of human rhinovirus in children with acute respiratory diseases in Chongqing China	No wheezing participants recruited
73	Lukkarinen, 2013	Prednisolone reduces recurrent wheezing after first rhinovirus wheeze: a 7-year follow-up	duplicate
74	Lukkarinen, 2014	Human bocavirus 1 may suppress rhinovirus-associated immune response in wheezing children	No data on viral etiology searched
75	Lukkarinen, 2016	The first rhinovirus-wheeze acts as a marker for later asthma in high-risk children	Correspondence
76	Lukkarinen, 2017	Rhinovirus-induced first wheezing episode predicts atopic but not nonatopic asthma at school age	duplicate
77	Lund, 2018	Atopic asthma after rhinovirus-induced wheezing is associated with DNA methylation change in the SMAD3 gene promoter	duplicate
78	Maffey, 2008	[New respiratory viruses in children 2 months to 3 years old with recurrent wheeze]	No PCR was used
79	Mahr, 2009	Wheezing Rhinovirus illnesses in early life predict asthma development in high-risk children.	Conference abstract
80	Mansbach, 2009	Respiratory viruses in bronchiolitis and their link to recurrent wheezing and asthma	Review
81	Matsuzaki, 2008	Clinical impact of human metapneumovirus genotypes and genotype-specific seroprevalence in Yamagata Japan	No wheezing participants recruited
82	Midulla, 2014	Recurrent wheezing 36 months after bronchiolitis is associated with rhinovirus infections and blood eosinophilia	No wheezing participants recruited
83	Mileva, 2018	Rhinovirus- induced wheezing in children with family history of asthma.	Conference abstract
84	Miller, 2009	Human rhinovirus C associated with wheezing in hospitalised children in the Middle East	No wheezing participants recruited
85	Miller, 2011	Atopy history and the genomics of wheezing after influenza vaccination in children 6-59 months of age.	No wheezing participants recruited
86	Mitchell I'Inglis, 1976	Viral infection in wheezy bronchitis and asthma in children	No wheezing participants recruited
87	Mitchell, 1978	Viral infection as a precipitant of wheeze in children Combined home and hospital study	No wheezing participants recruited
88	Moreno, 2009	[Respiratory tract infections and wheezing in children What role does rhinovirus play?]	Comment
89	Nenna, 2015	Viral Load in Infants Hospitalized for Respiratory Syncytial Virus Bronchiolitis Correlates with Recurrent Wheezing at Thirty-Six-Month Follow-Up	No wheezing participants recruited
90	Nieminen, 2013	Rhinovirus genotypes in the first wheeze.	Conference abstract
91	O'Callaghan-Gordo, 2013	Lower Respiratory Tract Infections Associated with Rhinovirus during Infancy and Increased Risk of Wheezing during Childhood A Cohort Study	No wheezing participants recruited
92	Okamoto, 2011	Increased eosinophilic cationic protein in nasal fluid in hospitalized wheezy infants with RSV infection	No wheezing participants recruited
93	Oo, 2015	Human rhinovirus species c has the greatest incidence in young children aged 2-10 years with acute severe wheeze.	Conference abstract
94	Openshaw, 2000	Protective and harmful effects of viral infections in childhood on wheezing disorders and asthma	Review

95	Osundwa, 1993	Recurrent wheezing in children with respiratory syncytial virus (RSV) bronchiolitis in Qatar	No wheezing participants recruited
96	Parkin, 2002	Controlled study of respiratory viruses and wheezing	No continuous study period
97	Pierangeli, 2013	Molecular epidemiology and genetic diversity of human rhinovirus affecting hospitalized children in Rome	No data on viral etiology searched
98	Pitrez, 2005	Inflammatory profile in nasal secretions of infants hospitalized with acute lower airway tract infections	No wheezing participants recruited
99	Puig, 2010	[Relationship between lower respiratory tract infections in the first year of life and the development of asthma and wheezing in children]	No data on viral etiology searched
100	Pullan, 1982	Wheezing asthma and pulmonary dysfunction 10 years after infection with respiratory syncytial virus in infancy	No wheezing participants recruited
101	Rakes, 1999	Rhinovirus and respiratory syncytial virus in wheezing children requiring emergency care IgE and eosinophil analyses	No continuous study period
102	Rattanadilok Na Bhuket, 2002	Wheezing--associated lower respiratory infections in under 5-year-old children: study in Takhli District Hospital	No wheezing participants recruited
103	Reijonen, 1997	Nasopharyngeal eosinophil cationic protein in bronchiolitis: relation to viral findings and subsequent wheezing	No wheezing participants recruited
104	Reijonen, 1997	Serum eosinophil cationic protein as a predictor of wheezing after bronchiolitis	No wheezing participants recruited
105	Reijonen, 1998	One-year follow-up of young children hospitalized for wheezing: the influence of early anti-inflammatory therapy and risk factors for subsequent wheezing and asthma	No data on viral etiology searched
106	Reijonen, 2000	Predictors of asthma three years after hospital admission for wheezing in infancy	No PCR was used
107	Renois, 2013	Enterovirus 68 in pediatric patients hospitalized for acute airway diseases	No wheezing participants recruited
108	Robertson, 2014	HRV-b and HRV-c species are more recurrent in children with acute wheeze and controls followed for 12 weeks.	Conference abstract
109	Romero, 2010	Serious early childhood wheezing after respiratory syncytial virus lower respiratory tract illness in preterm infants	Only participants with comorbidities
110	Rossi, 2015	Infantile respiratory syncytial virus and human rhinovirus infections: respective role in inception and persistence of wheezing	Review
111	Rubner, 2014	Viral etiology of early life wheezing illnesses differentially predict persistence of asthma in high-risk children.	Conference abstract
112	Rubner, 2017	Early life rhinovirus wheezing allergic sensitization and asthma risk at adolescence	No wheezing participants recruited
113	Rylander, 1988	Risk factors for occasional and recurrent wheezing after RSV infection in infancy	No data on viral etiology searched
114	Rylander, 1996	Wheezing bronchitis in children Incidence viral infections and other risk factors in a defined population	No PCR was used
115	Schauer, 2002	RSV bronchiolitis and risk of wheeze and allergic sensitisation in the first year of life	No wheezing participants recruited
116	Schildgen, 2006	Wheezing in patients with human metapneumovirus infection	Letter to editor

117	Schuez-Havupalo, 2014	Association between infant swimming and rhinovirus-induced wheezing	No wheezing participants recruited
118	Shilts, 2017	Respiratory syncytial virus (RSV) genotypes associated with wheezing in infants have distinct nasopharyngeal microbiome community structure during acute viral infection.	Conference abstract
119	Simões, 2010	The effect of respiratory syncytial virus on subsequent recurrent wheezing in atopic and nonatopic children	Only participants with comorbidities
120	Simons, 2005	Analysis of tracheal secretions for rhinovirus during natural colds	No wheezing participants recruited
121	Sims, 1981	Atopy does not predispose to RSV bronchiolitis or postbronchiolitic wheezing	No wheezing participants recruited
122	Smyth, 1999	Respiratory syncytial virus and wheeze	No data on viral etiology searched
123	Söderlund-Venermo, 2009	Clinical Assessment and Improved Diagnosis of Bocavirus-induced Wheezing in Children Finland	duplicate
124	Soto-Quiros, 2012	High titers of IgE antibody to dust mite allergen and risk for wheezing among asthmatic children infected with rhinovirus	No continuous study period
125	Stein, 1999	Respiratory syncytial virus in early life and risk of wheeze and allergy by age 13 years	No wheezing participants recruited
126	Stein, 2009	Long-term airway morbidity following viral LRTI in early infancy: recurrent wheezing or asthma?	Review
127	Stenberg Hammar, 2013	Relevance of respiratory infections in preschool wheeze-A hospital based study.	Conference abstract
128	Stenberg Hammar, 2018	Reduced CDHR3 expression in children wheezing with rhinovirus	duplicate
129	Stenberg-Hammar, 2015	Rhinovirus species and specific antibody response in preschool children with acute wheeze.	Conference abstract
130	Stoner, 2018	Rhinovirus causes increased acute symptoms in children with first time wheezing compared to either controls with cold symptoms or children with first time wheezing and Respiratory Syncytial Virus.	Conference abstract
131	Sugai, 2015	Respiratory syncytial virus and rhinovirus contribute to the first wheeze episodes in Japanese infants.	Conference abstract
132	Sumino, 2013	Hmpv bronchiolitis is associated with increased risk for subsequent wheezing in early life	Conference abstract
133	Suzuki, 2003	[Detection of human metapneumovirus from wheezing children in Japan]	No wheezing participants recruited
134	Tang, 2018	Analysis of the pathogens and clinical characteristics of acute wheezing disorders in children under 6 years old.	Complete text not found
135	Tapia, 2013	Respiratory syncytial virus infection and recurrent wheezing in Chilean infants: a genetic background?	No continuous study period
136	Tian, 2009	Effect of variation in RANTES promoter on serum RANTES levels and risk of recurrent wheezing after RSV bronchiolitis in children from Han Southern China	No data on viral etiology searched
137	Tortora, 2015	Adenovirus species C detection in children under four years of age with acute bronchiolitis or recurrent wheezing	No wheezing participants recruited
138	Turunen, 2016	Rhinovirus species and clinical characteristics in the first wheezing episode in children	duplicate
139	Turunen, 2016	12-month clinical and virus surveillance after the first wheezing episode: Special reference to rhinovirus-A and -C species.	Conference abstract

140	Turunen, 2017	Clinical and Virus Surveillance After the First Wheezing Episode: Special Reference to Rhinovirus A and C Species	duplicate
141	Tzocheva, 2017	Influence of respiratory viruses on the severity of bronchial obstruction in preschool wheezing children.	Conference abstract
142	Valkonen, 2009	Recurrent wheezing after respiratory syncytial virus or non-respiratory syncytial virus bronchiolitis in infancy: a 3-year follow-up	No continuous study period
143	van der Gugten, 2013	Reduced neonatal lung function and wheezing illnesses during the first 5 years of life	No data on viral etiology searched
144	van der Gugten, 2013	Human rhinovirus and wheezing: short and long-term associations in children	No wheezing participants recruited
145	Welliver, 1986	Predictive value of respiratory syncytial virus-specific IgE responses for recurrent wheezing following bronchiolitis	No continuous study period
146	Wennergren, 1997	Wheezing bronchitis reinvestigated at the age of 10 years	No data on viral etiology searched
147	Williams, 2005	Human metapneumovirus infection in children hospitalized for wheezing	No data on viral etiology searched
148	Wu, 2017	Clinical and epidemiological characteristics of human parainfluenza virus infections of children in southern Taiwan	No wheezing participants recruited
149	Xing, 2018	Differences in viral etiologies in asthma and wheezing illness in young children.	Conference abstract
150	Xu, 2017	Comparative Diagnosis of Human Bocavirus 1 Respiratory Infection With Messenger RNA Reverse-Transcription Polymerase Chain Reaction (PCR) DNA Quantitative PCR and Serology	No wheezing participants recruited
151	Yang, 2016	[Risk factors for recurrent wheezing in infants and young children suffering from dust mite allergy after their first wheezing]	No data on viral etiology searched
152	Yasuno, 2008	Wheezing illness caused by respiratory syncytial virus and other agents	No PCR was used
153	Younes, 1982	Wheezing asthma and pulmonary dysfunction 10 years after infection with respiratory syncytial virus in infancy	Letter to editor
154	Zakiul Hassan, 2017	Respiratory virus associated with wheezing in early life: A birth cohort study in a low-income urban community in Dhaka, Bangladesh.	Conference abstract
155	Zomer-Kooijker, 2014	Decreased lung function precedes severe respiratory syncytial virus infection and post-respiratory syncytial virus wheeze in term infants	No continuous study period
