# Supplementary material for: Prevalence of respiratory viruses using polymerase chain reaction in children with wheezing, a systematic review and meta–analysis
Source: PLoS One. 2020 Dec 14;15(12):e0243735. doi: 10.1371/journal.pone.0243735 (PMC7735590; doi:10.1371/journal.pone.0243735)
Supplement: S5 Table — (PDF) [file pone.0243735.s023.pdf]

S5 Table. Individual characteristics of included studies

Name, Year	Design	Country	Sampling	Period	Clinical_presentation	Case_definition	Detection assay	Sample_type	Age range; mean or median (years)	Male (%)	Bias	list of viruses studied	Rhinovirus (%)	HRSV (%)	HAdV (%)	Influenza (%)	HMPV (%)	HBoV (%)	HPIV (%)	Enterovirus (%)	HCoV (%)
Allander, 2007	Retrospective clinical trial	Finland	Random	Sep/2000-May/2002	Acute wheezing	Acute expiratory wheezing was considered to be bronchiolitis when it occurred for the first time in children aged <3 years. Asthma was diagnosed on the basis of the National Asthma Education and Prevention Program guidelines [14]. All other episodes of acute expiratory wheezing were considered to be recurrent wheezing.	Classical PCR	Nasopharyngeal aspirate; Nasal swab	0-18 Years ; 1,6	NR	low risk	HBoV						18,9			
Bedolla-Barajas, 2017	Prospective cross sectional	Mexico	Consecutive	Dec/2012-Apr/2013	Acute wheezing	Upon admission, the demographic characteristics and medical history of the children were systematically recorded, using standardized written questionnaires and, after a complete physical examination, children with a diagnosis of infectious wheezing, based on well-established criteria [28], were enrolled.	Classical PCR	Nasal swab	< 2 years ; NR	63,6	moderate risk	RV; HRSV; HAdV; Influenza; HMPV; HBoV; HPIV	12,7	12,7	10,9	5,5	7,2	14,5	5,5		
Bosis, 2008	Prospective cohort	Italy	Consecutive	Oct/2005-Mar/2006	Acute wheezing	Unclear/Not reported	Real-time PCR	Nasopharyngeal swab	< 2 years ; 0,2	67	low risk	RV; HRSV; HAdV; Influenza; HMPV; HBoV; HPIV; EV; HCoV	4,7	74,1	3,5	8,2	2,6	2,4	1,2	1,2	7,1
Camara, 2004	Prospective case-control	Brazil	Consecutive	Oct/1998-Jun/2000	Acute wheezing	Unclear/Not reported	Classical PCR	Nasal wash	0-18 Years ; NR	60,6	moderate risk	RV; HCoV	20,3								4,1
Chung, 2007	Prospective cross sectional	South Korea	Consecutive	Feb/2006-Nov/2006	Acute wheezing	Unclear/Not reported	Classical PCR	Nasopharyngeal aspirate	< 5 Years ; 0,7	62,3	moderate risk	RV; HMPV; HBoV	31,2				30	10			
Cox, 2013	Prospective cross sectional	Australia	Consecutive	Jul/2002-Nov/2010	Acute wheezing	Unclear/Not reported	Classical PCR	Nasopharyngeal aspirate	< 5 Years ; 2,6	62,9	moderate risk	RV	68,5								
de Winter, 2015	Prospective cohort	Netherlands	Consecutive	Jan/2006-Dec/2008	Acute wheezing	RV-WI in the first year of life was defined as the simultaneous presence of parent-reported wheezing for 2 days (based on daily logs) and molecular rhinovirus detection in viral samples collected during respiratory episodes in the first year of life [24]. The primary outcome of the study, medically attended third year wheezing, was defined as the use of prescribed inhaled asthma medications (corticosteroids and/or b2-ympathomimetics) together with a doctor¡¦s visit for respiratory symptoms in the third year of life. The secondary outcome, physician-diagnosed wheezing, was composed of a doctor¡¦s diagnosis of wheezing or the use of prescribed inhaled asthma medications (corticosteroids and/or b2-sympathomimetics) in the third year of life. A doctor¡¦s diagnosis of wheezing was defined according to the International Classification of Primary Care (ICPC) of either wheezing (R03) or asthma (R96).	Real-time PCR	Nasopharyngeal swab	< 2 years ; NR	51	low risk	RV	9,9								
Deng, 2007	Prospective cross sectional	China	Consecutive	Apr/2006-Jan/2007	Recurrent wheezing	Unclear/Not reported	Classical PCR	Nasopharyngeal aspirate, Tracheal aspirate	NR ; NR	NR	moderate risk	HBoV						30,8			
Fuenzalida, 2010	Prospective cross sectional	Spain	Consecutive	Oct/2006-Oct-2007	Acute wheezing	Unclear/Not reported	Real-time PCR	Nasopharyngeal aspirate	< 5 Years ; NR	NR	moderate risk	HMPV					21,1				
Fujitsuka, 2011	Prospective cross sectional	Japan	Consecutive	Nov/2007-Mar/2009	Acute wheezing	Unclear/Not reported	Classical PCR	Nasopharyngeal swab	NR ; 1,7	60,9	moderate risk	RV; HRSV; HAdV; Influenza; HMPV; HPIV; EV	40,9	53	0	0	1,2		0,9	0	
Garcia-Garcia, 2010	Prospective cross sectional	Spain	Consecutive	Sep/2005-Jun/2008	Acute wheezing	In order to reduce as much as possible the variability of the patients included, and according to the criteria of McConnochie,9 acute expiratory wheezing was considered to be bronchiolitis when it occurred for the first time in children aged <2 years and these children were, therefore excluded.	Classical PCR	Nasopharyngeal aspirate	0-18 Years ; NR	NR	low risk	RV; HRSV; HAdV; Influenza; HMPV; HBoV; HPIV; EV; HCoV	15,6	18,9	8,7	1,5	80,9	6,1	1,5	0,7	0,2
Halmo Hurdum, 2015	Prospective cohort	Australia	Consecutive	Aug/2002-Oct/2012	Acute wheezing	Unclear/Not reported	Classical PCR	Nasopharyngeal aspirate, Nasal wash	0-18 Years ; 4,5	60,1	moderate risk	RV	69,2								
Hancerli-Torun, 2015	Prospective cross sectional	Turkey	Consecutive	Sep/2013-May/2014	Acute wheezing	Unclear/Not reported	Real-time PCR	Nasopharyngeal swab, Tracheal aspirate	< 5 Years ; 0,9	61,9	moderate risk	RV; HRSV; HAdV; Influenza; HMPV; HBoV; HPIV; HCoV	13	33,7	3,3	16,3	4,6	3,3	1,1		5,4
Jartti, 2002	Prospective clinical trial	Finland	Consecutive	Sep/2000-May/2001	Acute wheezing	Unclear/Not reported	Classical PCR	Nasopharyngeal aspirate	0-18 Years ; 2	NR	moderate risk	HMPV					13,2				
Jartti, 2004	Prospective clinical trial	Finland	Random	Sep/2000-May/2002	Acute wheezing	Unclear/Not reported	Classical PCR	Nasopharyngeal aspirate	0-18 Years ; 1,6	NR	low risk	RV; HRSV; HMPV; EV; HCoV	22,3	17,9			34,9			20,3	1,4
Korppi, 2004	Retrospective clinical trial	Finland	Random	Jan/1992-Nov/1993	Acute wheezing	Unclear/Not reported	Classical PCR	Nasopharyngeal aspirate	< 2 years ; 6,3	74,3	low risk	RV; HRSV	33,3	30,9							
Kotaniemi-Syrjanen, 2003	Retrospective clinical trial	Finland	Random	Jan/1992-Nov/1993	Acute wheezing	Unclear/Not reported	Classical PCR	Nasopharyngeal aspirate	< 2 years ; 6,3	74,3	low risk	EV; HCoV								12,3	0
Lethbridge, 2018	Prospective cohort	Australia	Consecutive	Apr/2013-Jun/2013	Acute wheezing	Unclear/Not reported	Classical PCR	Nasal swab, Nasal-blow	NR ; NR	NR	moderate risk	RV	92,3								
Moattari, 2010	Prospective cross sectional	Iran	Consecutive	Sep/2008-May/2009	Acute wheezing	Unclear/Not reported	Classical PCR	Nasal swab	< 5 Years ; NR	NR	moderate risk	HMPV					24				
Mummidi, 2017	Prospective cross sectional	India	Consecutive	Aug/2013-Aug/2014	Acute wheezing	Viral wheeze (which was defined as wheezing occurring during discrete time periods with symptom-free period between episodes, usually associated with viral respiratory tract infection.	Real-time PCR	Nasal swab, Throat swab	< 5 Years ; 1,2	75	low risk	RV; HRSV; HBoV; HPIV	1,4	10				2,9	5,7		
Ong, 2007	Prospective cross sectional	Singapore	Random	Jan/2004-Jul/2005	Recurrent wheezing	Unclear/Not reported	Classical PCR	Nasopharyngeal aspirate	0-18 Years ; NR	NR	low risk	RV; HRSV; HAdV; Influenza; HMPV; HPIV	55	5	6,7	23,3	4,8		18,3		
Piotrowska, 2009	Prospective cross sectional	USA	Consecutive	Jan/2004-Dec/2004	Acute wheezing	Unclear/Not reported	Classical PCR	Nasopharyngeal aspirate	< 2 years ; NR	NR	moderate risk	RV	26,3								
Sackesen, 2005	Prospective cross sectional	Turkey	Consecutive	Feb/1999-Dec/1999	Recurrent wheezing	Unclear/Not reported	Classical PCR	Throat swab	NR ; 3,41	62,9	low risk	HAdV			63						
Shen, 2013	Prospective cross sectional	China	Consecutive	Dec/2012-Feb/2013	Acute wheezing	Unclear/Not reported	Classical PCR	Nasopharyngeal swab	0-18 Years ; NR	57,5	moderate risk	HBoV						5,5			
Smuts, 2008	Prospective cross sectional	South Africa	Consecutive	May/2004-Nov/2005	Acute wheezing	Unclear/Not reported	Classical PCR	Nasopharyngeal swab	0-18 Years ; 1	NR	moderate risk	HMPV; HBoV; HCoV					42,8	5,5			2,1
Smuts, 2011	Retrospective cross sectional	South Africa	Consecutive	May/2004-Nov/2005	Acute wheezing	Unclear/Not reported	Classical PCR	Nasal swab	< 5 Years ; 1	NR	moderate risk	RV	58,2								
Stenberg-Hammar, 2016	Prospective cohort	Sweden	Consecutive	Oct/2008-Sep/2012	Acute wheezing	Unclear/Not reported	Classical PCR	Nasopharyngeal swab	< 5 Years ; 1,5	71,2	moderate risk	RV	74,1								
Sun, 2016	Prospective cohort	China	Consecutive	Dec/2012-Nov/2014	Acute wheezing	Unclear/Not reported	Classical PCR	Nasopharyngeal samples	< 5 Years ; NR	70,5	moderate risk	RV; HMPV; HBoV	14,7				21,3	8			
Takeyama, 2014	Prospective cohort	Japan	Consecutive	Feb/2008-Aug/2009	Acute wheezing	Unclear/Not reported	Real-time PCR	Nasopharyngeal aspirate	< 5 Years ; NR	NR	moderate risk	RV; HRSV; Influenza; HMPV; HPIV	21,6	41,2		3,3	7,7		3,3		
Teeratakulpisarn, 2014	Prospective clinical trial	Thailand	Random	Apr/2002-Aug/2004	Acute wheezing	Recurrent wheezing was defined as children with a history of healthcare visits with wheezing or having received ƒÒ2 agonist nebulization to relieve respiratory symptoms.	Real-time PCR	Nasopharyngeal secretion	< 2 years ; 0,8	62,9	low risk	RV; HRSV; Influenza	18,2	64,7		17,6					
Turunen, 2014	Prospective clinical trial	Finland	Random	Jun/2007-Mar/2009	Acute wheezing	Wheezing was defined as a high-pitched whistling sound in expiration with breathing difficulty (1).	Classical PCR	Nasopharyngeal aspirate	< 2 years ; 1	67	low risk	RV; HRSV; HAdV; Influenza; HMPV; HPIV; EV; HCoV	75,7	28,8	3,6	1,8	7,8		9	3,6	4,5
van der Schee, 2015	Prospective cohort	Netherlands	Random	Dec/2010-Dec/2012	Acute wheezing	Unclear/Not reported	Real-time PCR	Nasal swab; Oropharyngeal swab	< 2 years ; 1,5	64,1	low risk	RV	45,7								
van der Zalm, 2011	Prospective cohort	Netherlands	Consecutive	Unclear/Not reported	Acute wheezing	wheezing is ¡¥¡¥a whistling sound from the chest and not from the nose or throat¡¦¡¦.	Classical PCR	Nasal swab, Throat swab	< 2 years ; 9	50,1	low risk	RV	83,3								
