# Supplementary material for: Prevalence of respiratory viruses using polymerase chain reaction in children with wheezing, a systematic review and meta–analysis
Source: PLoS One. 2020 Dec 14;15(12):e0243735. doi: 10.1371/journal.pone.0243735 (PMC7735590; doi:10.1371/journal.pone.0243735)
Supplement: S6 Table — (PDF) [file pone.0243735.s024.pdf]

S6 Table. Subgroup prevalence of respiratory viral infections in people with wheezing

	Prevalence. % (95%CI)	95% Prediction interval	N Studies	N Participants	H (95%CI)	I^2 (95%CI)	P heterogeneity	P Egger test	P difference subtypes
**Rhinovirus**									
**Age, mean or median, years**									0.024
< 1 year	31.3 [12.8-53.5]	[0-98.4]	6	909	6.8 [5.6-8.4]	97.9 [96.8-98.6]	< 0.001	0.501	
1-2 years	33.8 [12.5-59.3]	[0-100]	5	665	6.4 [5.1-8.1]	97.6 [96.1-98.5]	< 0.001	0.788	
2-18 years	64.9 [48.5-79.7]	[1.7-100]	4	956	5 [3.7-6.9]	96 [92.5-97.9]	< 0.001	0.221	
**WHO_Region**									< 0.001
Africa	58.2 [51.6-64.6]	NA	1	220	NA	NA	1	NA	
America	19.9 [13-27.9]	[0-97.1]	3	209	1.4 [1-2.5]	45.3 [0-83.8]	0.161	0.168	
Europe	35.8 [16.9-57.2]	[0-99.4]	10	1874	9.2 [8.1-10.4]	98.8 [98.5-99.1]	< 0.001	0.712	
South-East Asia	8.2 [0-30.8]	NA	2	240	4.2 [2.4-7.5]	94.4 [82.7-98.2]	< 0.001	NA	
Western Pacific	47.9 [28-68.1]	[0-100]	8	1851	8.3 [7.1-9.6]	98.5 [98-98.9]	< 0.001	0.285	
**Detection assay**									< 0.001
Classical PCR	45.8 [31.9-60.1]	[0.5-97.3]	17	3562	8.4 [7.6-9.3]	98.6 [98.3-98.8]	< 0.001	0.216	
Real-time PCR	14.3 [6.7-24.1]	[0-54.7]	7	832	3.6 [2.7-4.7]	92.2 [86.4-95.5]	< 0.001	0.803	
**HRSV**									
**Age, mean or median, years**									0.221
< 1 year	50.4 [29-71.7]	[0-100]	4	458	4.7 [3.4-6.5]	95.5 [91.3-97.7]	< 0.001	0.738	
1-2 years	25.3 [6.3-51.2]	[0-100]	3	464	5.4 [3.7-7.7]	96.5 [92.8-98.3]	< 0.001	0.869	
2-18 years	30.9 [21.2-41.4]	NA	1	81	NA	NA	1	NA	
**WHO_Region**									0.098
America	12.7 [5-23]	NA	1	55	NA	NA	1	NA	
Europe	33 [19.9-47.6]	[0.2-83.9]	6	1187	4.8 [3.7-6.2]	95.6 [92.8-97.4]	< 0.001	0.092	
South-East Asia	34.9 [0-88.9]	NA	2	240	8.5 [5.8-12.5]	98.6 [97.1-99.4]	< 0.001	NA	
Western Pacific	30.6 [8.2-59.4]	[0-100]	3	328	5.2 [3.6-7.6]	96.3 [92.3-98.3]	< 0.001	0.373	
**Detection assay**									0.07
Classical PCR	22.7 [14.1-32.8]	[0.7-60.7]	7	1240	3.6 [2.8-4.8]	92.4 [86.9-95.6]	< 0.001	0.702	
Real-time PCR	43.9 [23.5-65.5]	[0-100]	5	570	5.2 [4-6.8]	96.3 [93.7-97.8]	< 0.001	0.42	
**HAdV**									
**Age, mean or median, years**									< 0.001
< 1 year	3.5 [1.5-6]	[0-29.2]	3	288	1 [1-1]	0 [0-0]	0.993	0.905	

	Prevalence. % (95%CI)	95% Prediction interval	N Studies	N Participants	H (95%CI)	I² (95%CI)	P heterogeneity	P Egger test	P difference subtypes
1-2 years	0 [0-1.5]	NA	1	115	NA	NA	1	NA	
2-18 years	63 [43.8-80.4]	NA	1	27	NA	NA	1	NA	
**WHO_Region**									0.319
America	10.9 [3.8-20.7]	NA	1	55	NA	NA	1	NA	
Europe	10.6 [3.1-21.5]	[0-61.2]	5	854	3.6 [2.6-5]	92.3 [85.1-96.1]	< 0.001	0.644	
Western Pacific	1.9 [0-13.3]	NA	2	175	2.9 [1.5-5.6]	87.9 [53.3-96.9]	0.004	NA	
**Detection assay**									0.119
Classical PCR	9.9 [2.6-20.8]	[0-57.9]	6	907	3.8 [2.9-5.1]	93.2 [87.9-96.2]	< 0.001	0.653	
Real-time PCR	3.4 [1.1-6.7]	NA	2	177	1 NA	0 NA	0.916	NA	
**Influenza**									
**Age, mean or median, years**									0.001
< 1 year	10 [3.2-19.6]	[0-67.1]	4	458	2.9 [1.9-4.5]	88.2 [72.3-95]	< 0.001	0.605	
1-2 years	0 [0-1.5]	NA	1	115	NA	NA	1	NA	
**WHO_Region**									0.016
America	5.5 [0.7-13.4]	NA	1	55	NA	NA	1	NA	
Europe	5.5 [0.8-13.4]	[0-59.3]	4	827	3.3 [2.2-4.9]	90.9 [79.9-95.9]	< 0.001	0.206	
South-East Asia	17.6 [12.3-23.8]	NA	1	170	NA	NA	1	NA	
Western Pacific	5.4 [0-20.6]	[0-100]	3	328	4.1 [2.7-6.3]	94.1 [86.2-97.5]	< 0.001	0.467	
**Detection assay**									0.152
Classical PCR	3.8 [0.3-10.2]	[0-38.7]	5	880	3.2 [2.2-4.5]	89.9 [79.4-95.1]	< 0.001	0.328	
Real-time PCR	10.5 [4.1-19.4]	[0-62.2]	4	500	2.8 [1.8-4.3]	87 [68.6-94.6]	< 0.001	0.956	
**HMPV**									
**Age, mean or median, years**									0.286
< 1 year	6 [4.3-7.8]	[3.4-9.1]	5	757	1 [1-1.5]	0 [0-53.8]	0.773	0.293	
1-2 years	3.8 [1.1-7.8]	[0-86.3]	3	538	1.9 [1-3.5]	72.8 [8.3-91.9]	0.025	0.894	
**WHO_Region**									< 0.001
Africa	7.6 [4.5-11.3]	NA	1	238	NA	NA	1	NA	
America	23.6 [13.2-35.9]	NA	1	55	NA	NA	1	NA	
Eastern mediterranean	16.7 [10.5-23.9]	NA	1	120	NA	NA	1	NA	
Europe	4.9 [3.3-6.9]	[1.1-10.9]	7	1412	1.4 [1-2.2]	50.6 [0-79]	0.059	0.056	
Western Pacific	3.3 [0.5-8]	[0-28.5]	5	1268	3.2 [2.2-4.5]	89.9 [79.4-95.1]	< 0.001	0.107	
**Detection assay**									0.573
Classical PCR	6.3 [3.2-10.3]	[0-25.1]	11	2601	3.5 [2.8-4.3]	91.7 [87.2-94.7]	< 0.001	0.003	

	Prevalence. % (95%CI)	95% Prediction interval	N Studies	N Participants	H (95%CI)	I² (95%CI)	P heterogeneity	P Egger test	P difference subtypes
Real-time PCR	5.1 [3-7.5]	[0.4-13.6]	4	492	1.1 [1-2.9]	19.9 [0-87.7]	0.29	0.713	
**HBoV**									
**Age, mean or median, years**									0.54
< 1 year	5.4 [2.6-8.9]	[0-24.4]	4	646	1.7 [1-2.9]	64.3 [0-87.9]	0.039	0.239	
1-2 years	9.8 [0.1-30.1]	NA	2	329	3.9 [2.2-7.1]	93.5 [78.9-98]	< 0.001	NA	
**WHO_Region**									0.074
Africa	5.5 [2.9-8.8]	NA	1	238	NA	NA	1	NA	
America	14.5 [6.3-25.3]	NA	1	55	NA	NA	1	NA	
Europe	6.9 [1.8-14.8]	[0-58.1]	4	975	3.6 [2.4-5.2]	92.2 [83.2-96.4]	< 0.001	0.805	
South-East Asia	2.9 [0-8.4]	NA	1	70	NA	NA	1	NA	
Western Pacific	10.1 [5.7-15.5]	[0-39.8]	4	1254	2.5 [1.6-3.9]	83.7 [58.6-93.5]	< 0.001	0.26	
**Detection assay**									0.001
Classical PCR	10.2 [6.8-14.2]	[1.2-25.8]	8	2345	2.8 [2-3.7]	86.8 [76.1-92.7]	< 0.001	0.142	
Real-time PCR	2.8 [1-5.4]	[0-30.5]	3	247	1 [1-1]	0 [0-0]	0.947	0.946	
**HPIV**									
**Age, mean or median, years**									0.894
< 1 year	3.1 [0.1-9.2]	[0-100]	3	288	2.1 [1.2-3.8]	78.3 [30-93.3]	0.01	0.198	
1-2 years	2.6 [0-9.3]	NA	2	185	1.9 [1-4]	71.6 [0-93.6]	0.061	NA	
**WHO_Region**									0.606
America	5.5 [0.7-13.4]	NA	1	55	NA	NA	1	NA	
Europe	2.5 [0.4-6.1]	[0-27]	4	827	2.1 [1.3-3.5]	77.3 [38.1-91.6]	0.004	0.55	
South-East Asia	5.7 [1.3-12.6]	NA	1	70	NA	NA	1	NA	
Western Pacific	5.4 [0.2-15.9]	[0-100]	3	328	3 [1.8-5]	89.2 [70.5-96]	< 0.001	0.395	
**Detection assay**									0.287
Classical PCR	5.3 [1.1-11.9]	[0-38.9]	5	880	2.9 [2-4.3]	88.5 [75.8-94.5]	< 0.001	0.14	
Real-time PCR	2.5 [1-4.6]	[0-9.4]	4	400	1.1 [1-2.8]	13.8 [0-86.8]	0.323	0.955	
**Enterovirus**									
**Age, mean or median, years**									0.012
< 1 year	2.4 [0.6-5.2]	NA	2	196	1 NA	0 NA	0.324	NA	
1-2 years	6.3 [0-38.7]	NA	2	406	7.7 [5.1-11.5]	98.3 [96.2-99.2]	< 0.001	NA	
2-18 years	12.3 [6-20.5]	NA	1	81	NA	NA	1	NA	
**WHO_Region**									0.042

	Prevalence. % (95%CI)	95% Prediction interval	N Studies	N Participants	H (95%CI)	I² (95%CI)	P heterogeneity	P Egger test	P difference subtypes
Europe	5.9 [0.3-16.9]	[0-64.7]	5	1107	5.4 [4.2-7]	96.6 [94.3-98]	< 0.001	0.658	
Western Pacific	0 [0-1.5]	NA	1	115	NA	NA	1	NA	
**Detection assay**									0.327
Classical PCR	5.1 [0.1-15.9]	[0-64.6]	5	1137	5.7 [4.4-7.3]	96.9 [94.8-98.1]	< 0.001	0.729	
Real-time PCR	1.2 [0-5]	NA	1	85	NA	NA	1	NA	
**HCoV**									
**Age, mean or median, years**									0.016
< 1 year	4.1 [2-6.9]	[0-16.8]	4	526	1.3 [1-2.3]	42.9 [0-80.8]	0.154	0.01	
1-2 years	1.4 [0.3-3.1]	NA	1	291	NA	NA	1	NA	
2-18 years	0 [0-2.1]	NA	1	81	NA	NA	1	NA	
**WHO_Region**									0.594
Africa	2.1 [0.6-4.4]	NA	1	238	NA	NA	1	NA	
America	4.1 [0.5-10.2]	NA	1	73	NA	NA	1	NA	
Europe	2.1 [0.3-5.1]	[0-16.4]	6	1199	2.5 [1.7-3.6]	83.6 [65.7-92.1]	< 0.001	0.076	
**Detection assay**									0.007
Classical PCR	1.4 [0.2-3.2]	[0-9.4]	6	1333	2 [1.3-3]	74.2 [41.2-88.7]	0.002	0.155	
Real-time PCR	6.2 [3-10.4]	NA	2	177	1 NA	0 NA	0.661	NA	

CI: confidence interval; RV: Rhinovirus; HCoV: Human Coronavirus; HPIV: Human Parainfluenzavirus; HMPV: Human Metapneumovirus; HRSV: Human Respiratory Syncytial Virus; HAdV: Human Adenovirus; HBoV: Human Bocavirus; EV: Enterovirus; NA: not applicable.
