# Supplementary material for: Prevalence of respiratory viruses using polymerase chain reaction in children with wheezing, a systematic review and meta–analysis
Source: PLoS One. 2020 Dec 14;15(12):e0243735. doi: 10.1371/journal.pone.0243735 (PMC7735590; doi:10.1371/journal.pone.0243735)
Supplement: S7 Table — (PDF) [file pone.0243735.s025.pdf]

S7 Table. Univariable and multivariable metaregression analysis on the prevalence of Respiratory Viruses in people with wheezing disorders.

Virus	Bivariate Model				Multivariate Model			
	Estimate	P-Value	P-Value Global	OR(95% CI)	Estimate	P-Value	OR [95% CI]	R2
**Rhinovirus (28 Studies)**								**10,77%**
**Year**			0,579					
2004	0			1				
2007 - 2018	0,008	0,579		1,01 [0,98 - 1,04]				
**Age, mean or median, years**			**0,082**		[redacted]			
< 1 year	0			1				
1-2 years	0,028	0,856		1,03 [0,76 - 1,39]				
2-18 years	0,338	**0,036**		1,4 [1,02 - 1,92]				
**WHO_Region**			0,268					
Africa	0			1				
America	-0,404	0,261		0,67 [0,33 - 1,35]				
Europe	-0,225	0,491		0,8 [0,42 - 1,51]				
South-East Asia	-0,572	**0,133**		0,56 [0,27 - 1,19]				
Western Pacific	-0,102	0,758		0,9 [0,47 - 1,72]				
**Detection assay**			**0,005**					
Classical PCR	0			1	0		1	
Real-time PCR	-0,351	**0,005**		0,7 [0,55 - 0,9]	-0,351	**0,005**	0,7 [0,55 - 0,9]	
**HRSV (14 Studies)**								**30,93%**
**Year**			0,982					
2004	0			1				
2007 - 2018	0	0,982		1 [0,97 - 1,03]				
**Age, mean or median, years**			0,312					
< 1 year	0			1				
1-2 years	-0,26	**0,137**		0,77 [0,55 - 1,09]				
2-18 years	-0,198	0,443		0,82 [0,5 - 1,36]				
**WHO_Region**			0,801					
America	0			1				
Europe	0,24	0,341		1,27 [0,78 - 2,09]				
South-East Asia	0,264	0,355		1,3 [0,74 - 2,28]				
Western Pacific	0,215	0,426		1,24 [0,73 - 2,1]				
**Detection assay**			**0,038**					
Classical PCR	0			1	0		1	
Real-time PCR	0,227	**0,038**		1,25 [1,01 - 1,56]	0,227	**0,038**	1,25 [1,01 - 1,56]	
**HAdV (9 Studies)**								**100,00%**
**Age, mean or median, years**			**0**					
< 1 year	0			1	0		1	
1-2 years	-0,153	**0,005**		0,86 [0,77 - 0,96]	-0,153	**0,005**	0,86 [0,77 - 0,96]	
2-18 years	0,712	**0**		2,04 [1,68 - 2,48]	0,712	**0**	2,04 [1,68 - 2,48]	
**WHO_Region**			0,4					
America	0			1				
Europe	-0,008	0,967		0,99 [0,69 - 1,43]				
Western Pacific	-0,191	0,358		0,83 [0,55 - 1,24]				
**Detection assay**			0,354					
Classical PCR	0			1				
Real-time PCR	-0,13	0,354		0,88 [0,67 - 1,16]				
**Influenza (10 Studies)**								**90,32%**
**Year**			0,797					
2007	0			1				
2008 - 2017	-0,005	0,797		1 [0,96 - 1,03]				
**Age, mean or median, years**			**0,068**					
< 1 year	0			1	0		1	
1-2 years	-0,281	**0,068**		0,76 [0,56 - 1,02]	-0,1027	0,3057	0,9 [0,74 - 1,1]	
**WHO_Region**			0,735					
America	0			1				
Europe	-0,007	0,972		0,99 [0,69 - 1,43]				
South-East Asia	0,184	0,428		1,2 [0,76 - 1,89]				
Western Pacific	-0,01	0,958		0,99 [0,68 - 1,44]				
**Detection assay**			**0,156**					
Classical PCR	0			1			1	
Real-time PCR	0,128	**0,156**		1,14 [0,95 - 1,36]	0,2417	**0,0033**	1,27 [1,08 - 1,5]	
**HMPV (19 Studies)**								
**Year**			0,812					
2002	0			1				
2004 - 2017	-0,002	0,812		1 [0,99 - 1,01]				

**Age, mean or median, years**			0,2					
< 1 year	0			1				
1-2 years	-0,046	0,2		0,96 [0,89 - 1,03]				
**WHO_Region**			**0,007**					
Africa	0			1				
America	0,231	**0,073**		1,26 [0,98 - 1,62]				
Eastern mediterranean	0,142	0,232		1,15 [0,91 - 1,46]				
Europe	-0,046	0,596		0,96 [0,8 - 1,13]				
Western Pacific	-0,095	0,286		0,91 [0,76 - 1,08]				
**Detection assay**			0,694					
Classical PCR	0			1				
Real-time PCR	-0,026	0,694		0,97 [0,86 - 1,11]				
**HBoV (13 Studies)**								**14,91%**
**Year**			**0,184**					
2007	0			1				
2008 - 2017	-0,01	**0,184**		0,99 [0,98 - 1,01]				
**Age, mean or median, years**			0,273					
< 1 year	0			1				
1-2 years	0,099	0,273		1,1 [0,92 - 1,32]				
**WHO_Region**			0,591					
Africa	0			1				
America	0,16	0,306		1,17 [0,86 - 1,6]				
Europe	0,034	0,769		1,03 [0,83 - 1,3]				
South-East Asia	-0,052	0,734		0,95 [0,7 - 1,28]				
Western Pacific	0,099	0,392		1,1 [0,88 - 1,38]				
**Detection assay**			**0,017**					
Classical PCR	0			1	0		1	
Real-time PCR	-0,144	**0,017**		0,87 [0,77 - 0,97]	-0,144	**0,017**	0,87 [0,77 - 0,97]	
**HPIV (10 Studies)**								
**Year**			0,898					
2007	0			1				
2008 - 2017	-0,001	0,898		1 [0,98 - 1,02]				
**Age, mean or median, years**			0,901					
< 1 year	0			1				
1-2 years	-0,012	0,901		0,99 [0,82 - 1,2]				
**WHO_Region**			0,816					
America	0			1				
Europe	-0,08	0,55		0,92 [0,71 - 1,2]				
South-East Asia	0,002	0,99		1 [0,72 - 1,4]				
Western Pacific	-0,012	0,931		0,99 [0,75 - 1,29]				
**Detection assay**			0,379					
Classical PCR	0			1				
Real-time PCR	-0,065	0,379		0,94 [0,81 - 1,08]				
**Enterovirus (7 Studies)**								
**Age, mean or median, years**			0,789					
< 1 year	0			1				
1-2 years	0,094	0,696		1,1 [0,69 - 1,75]				
2-18 years	0,2	0,499		1,22 [0,68 - 2,18]				
**WHO_Region**			0,35					
Europe	0			1				
Western Pacific	-0,205	0,35		0,81 [0,53 - 1,25]				
**Detection assay**			0,646					
Classical PCR	0			1				
Real-time PCR	-0,104	0,646		0,9 [0,58 - 1,4]				
**HCoV (9 Studies)**								**53,45%**
**Year**			0,325					
2003	0			1				
2004 - 2015	0,008	0,325		1,01 [0,99 - 1,02]				
**Age, mean or median, years**			**0,052**					
< 1 year	0			1	0		1	
1-2 years	-0,09	**0,117**		0,91 [0,82 - 1,02]	-0,09	**0,117**	0,91 [0,82 - 1,02]	
2-18 years	-0,159	**0,032**		0,85 [0,74 - 0,99]	-0,159	**0,032**	0,85 [0,74 - 0,99]	
**WHO_Region**			0,852					
Africa	0			1				
America	0,067	0,631		1,07 [0,81 - 1,4]				
Europe	0,005	0,957		1,01 [0,83 - 1,22]				
**Detection assay**			**0,033**					
Classical PCR	0			1				
Real-time PCR	0,131	**0,033**		1,14 [1,01 - 1,29]				
