# Supplementary material for: Prevalence of respiratory viruses using polymerase chain reaction in children with wheezing, a systematic review and meta–analysis
Source: PLoS One. 2020 Dec 14;15(12):e0243735. doi: 10.1371/journal.pone.0243735 (PMC7735590; doi:10.1371/journal.pone.0243735)
Supplement: Attachment — Submitted filename: PONE-D-20-03613_Comments due 07 July 2020.docx [file pone.0243735.s026.docx]

PONE-D-20-03613	Accepted June 27 2020

Due July 07 2020

Submitted July 04 2020

Prevalence of respiratory viruses using molecular detection in children with wheezing, a systematic review and meta–analysis

Comments:

LINE numbers would have facilitated speed of the review.

Spacings and superscript issues dot this manuscript. Please fix.

Figure 2.

Is there a way to simplify the items in Figure 2? It looks like a hybrid between a forest plot and table and would the authors express the significance in terms of the p-value?

If random-effects were used throughout not necessary to repeat the term

It seems that heterogeneity is substantial did the authors take steps to adjust for heterogeneity?

Were sources of heterogeneity identified?

PAGE 8

The studies were published between 2002 and 2018. Most were cross–sectional studies (16; 48.5%),

“Most” is followed by “48.5%”. This is under 50%, how could it be most? Did I miss something here?

Table 1. Sociodemographic and clinical characteristics of included studies

Age in years is expressed in median IQR, why not mean SD? Did the authors perform test for normal distribution, if so, please show the results.

Table 2. Overall meta-analysis for global prevalence of respiratory viral infections in children with wheezing

1.6. Supplemental table 6. Subgroup prevalence of respiratory viral infections in people with wheezing

The authors generated several prevalence values with indications of their significance or its absence. Did the authors perform corrections for multiple comparisons?
